# Amoeba‐Inspired Soft Robot for Integrated Tumor/Infection Therapy and Painless Postoperative Drainage

**DOI:** 10.1002/advs.202407148

**Published:** 2024-11-04

**Authors:** Wanyi Zhou, Peizheng Xiong, Yiman Ge, Yuhan He, Yue Sun, Gang Zhang, Yifan Chen, Chunhui Wu, Wei Zhang, Yiyao Liu, Hong Yang

**Affiliations:** ^1^ Department of Orthopedics Sichuan Provincial People's Hospital School of Life Science and Technology University of Electronic Science and Technology of China Chengdu Sichuan 610054 P. R. China; ^2^ TCM Regulating Metabolic Diseases Key Laboratory of Sichuan Province Hospital of Chengdu University of Traditional Chinese Medicine No. 39 Shi‐er‐qiao Road Chengdu Sichuan 610072 P. R. China; ^3^ School of Mechanical and Electrical Engineering Chengdu University of Technology Chengdu Sichuan 610059 P. R. China; ^4^ Department of Oncology Chengdu Second People's Hospital Chengdu Sichuan 610072 P. R. China; ^5^ Department of Urology Deyang People's Hospital Deyang Sichuan 618099 P. R. China; ^6^ Chongqing Engineering Laboratory of Nano/Micro Biomedical Detection Technology Chongqing University of Science and Technology Chongqing 401331 P. R. China

**Keywords:** hydrogel dressing, painless drainage, soft robot, tumor/infection therapy

## Abstract

Tumor recurrence and wound infection are devastating complications of wide excision surgery for melanoma, and deep postoperative wound drainage typically increases pain. An amoeba‐inspired magnetic soft robot (ASR) with switchable dormant and active phases is developed to address the aforementioned challenges. The dormant ASR supports wounds through its solid‐like elasticity and regulates reactive oxygen species (ROS) levels bidirectionally, promoting healing in infected wounds and eliminating residual tumors. It solves the challenge caused by the contradictory need for ROS scavenging in wound healing and ROS amplification in tumor/infection management. The active ASR removes absorbed wound exudate by crawling out from irregular wounds; interestingly, this crawling motion prevents damage to fragile tissues and alleviates wound pain via “non‐direct friction.” More importantly, ASR switches different states in response to an alternating magnetic field owing to its magnetothermal properties, and this process also exerts synergistic antitumor and bacteriostatic effects. Due to the appropriate mechanical structure (cohesive force) of ASR, the content of magnetic nanoparticles required to drive the ASR is ten‐fold lower than that of conventional magnetic soft robots, enabling in vivo degradation. These outcomes highlight the vantage of the ASR for treating post‐tumor excision wounds and underscore their potential for clinical application.

## Graphical abstract

The article presents an amoeba‐inspired soft robot designed for painless postoperative drainage. The robot supports wounds and bi‐directionally regulates reactive oxygen species during its dormant phase. In its active phase, it painlessly removes absorbed pus by crawling out of wounds with “non‐direct friction.” This robot shows promising therapeutic effects on wounds after tumor removal.

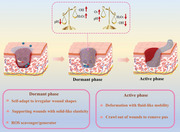

## 1 Introduction

The primary clinical approach for treating malignant melanoma is wide excision surgery, which involves the removal of the tumor along with at least 2 cm of surrounding soft tissue.^[^ ^1^ ^]^ However, postoperative tumor recurrence and wound infection are devastating complications of melanoma surgery.^[^ ^2^, ^3^ ^]^ Additionally, fat liquefaction and abscess formation can also delay the healing of surgical incisions and require management through repeated wound exudate drainage and dressing changes.^[^ ^4^ ^]^ Therefore, the development of a dressing that can prevent tumor recurrence, inhibit bacterial invasion, and rapidly remove wound exudate is urgently needed.

Hydrogels accelerate healing by keeping the wound moist and absorbing tissue exudate, which has been of considerable interest.^[^ ^5^ ^]^ In situ gelling hydrogel dressings, such as injectable preformed hydrogels and in situ polymerizing hydrogels, create the best wound barriers by adapting to irregularly shaped wounds.^[^ ^6^ ^]^ Nevertheless, these hydrogels that tightly adhere to tissues are rigid and challenging to remove from deep wounds. To alleviate secondary wound damage during dressing changes, two key strategies have been adopted. The first strategy involves the accelerated in situ absorbable degradation of the dressings.^[^ ^7^, ^8^, ^9^ ^]^ The second strategy activates the gel‐sol transition of the dressing and a negative pressure device is employed to suction the liquefied gel and exudate from deep wounds.^[^ ^10^, ^11^, ^12^ ^]^ However, wound dressings that undergo degradation in situ cannot discharge exudate promptly, increasing the risk of poor wound healing. Further, a sustained suction force at the wound site can aggravate pain. Although other types of dressings such as gauzes, hydrocolloids, shape‐fixed hydrogels, and degradable sponges have been developed, they also show similar limitations.^[^ ^13^ ^]^ These dressings often fail to self‐adapt to ever‐changing wounds due to their inability to reshape in irregular deep wounds, and cannot achieve painless drainage.

Hydrogel soft robots that share mechanical properties with human soft tissues have recently emerged as potential clinical tools.^[^ ^14^ ^]^ Owing to their flexibility, these soft robots can perform multiple types of motion and adapt to complex environments. Thus, we hypothesized that a hydrogel soft robot that can self‐adapt to irregular wounds and carry absorbed exudate out of wounds without causing pain and tissue damage may solve the problems caused by fixed wound dressings. Unfortunately, to our knowledge, a soft robot that serves as a wound dressing is yet to be developed.

In addition to enabling painless postoperative drainage, an ideal hydrogel robot should also self‐renew therapeutic programs by responding to the wound microenvironment, dynamically promoting wound healing. Reactive oxygen species (ROS) elevation is recognized as a powerful weapon for killing residual tumor cells and bacteria in surgical wounds after melanoma resection, this elevation can be achieved by chemodynamic therapy, photodynamic therapy, and increased endogenous ROS production.^[^ ^15^, ^16^, ^17^ ^]^ Such as peroxidase (POD)‐like nanozymes, which catalyze the production of hydroxyl radicals (•OH) from hydrogen peroxide (H_2_O_2_) via the Fenton reaction to induce intracellular ROS accumulation.^[^ ^15^ ^]^ However, ROS acts as a double‐edged sword, and evidence indicates that reducing ROS levels is critical for wound healing after melanoma excision surgery.^[^ ^18^, ^19^, ^20^ ^]^ Excessive ROS can cause undesirable inflammation, hindering wound healing and further promoting tumor recurrence, deterioration, and metastasis.^[^ ^21^, ^22^ ^]^ To solve the contradictory dilemma surrounding the need for decreased ROS in wound healing and increased ROS for tumor/infection management, a hydrogel robot should have the capacity to switch between ROS production and ROS scavenging depending on the dynamic demand for ROS within the wound microenvironment.

**Scheme 1:**
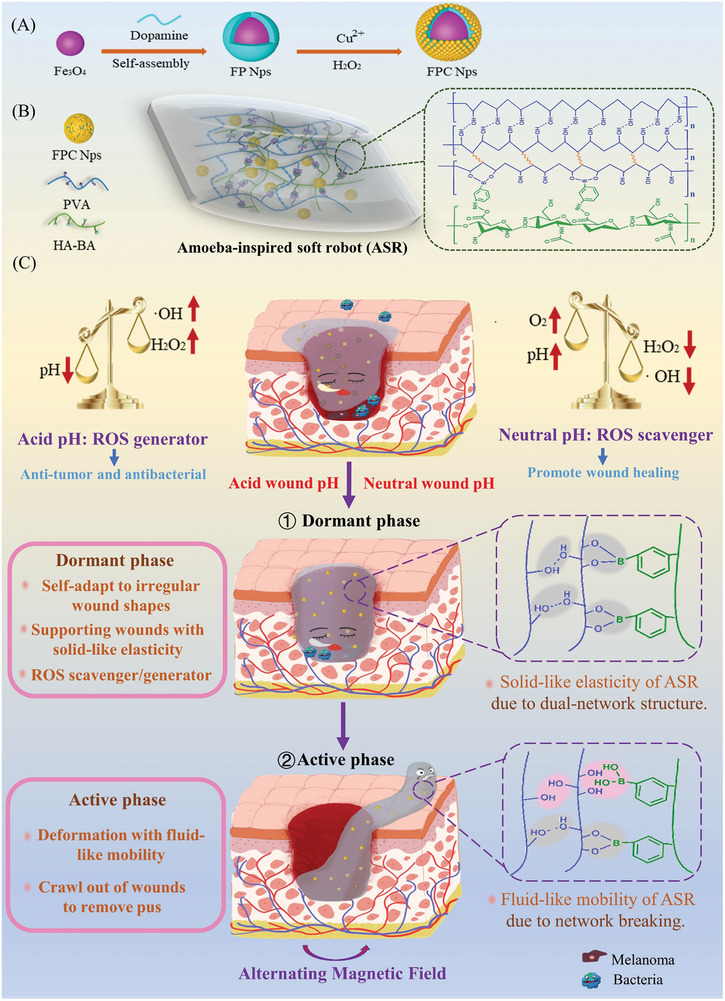
Design strategy for the amoeba‐inspired soft robot. (A) Preparation of FPC Nps. (B) Preparation of ASR. (C) The process of treating surgical wounds after melanoma resection.

Amoeba, as a single‐cell organism, is dormant at low temperatures and active at ambient temperatures, deforming and moving freely. In this study, we developed an amoeba‐inspired hydrogel soft robot (ASR) with switchable dormant and active phases (**Scheme** **1**). The dormant ASR self‐adapted to irregular wounds and supported them with solid‐like elasticity. It also enabled on‐demand ROS amplification/scavenging to achieve a dynamic balance among the antitumor, antibacterial, and wound‐healing processes. In contrast, the active ASR had greater amoeba‐like mobility. Under the exogenous control of a permanent magnet, it could deform and crawl out of wounds to remove absorbed exudate. Interestingly, ASR crawled away from the fragile new tissue via a “non‐direct friction” process, preventing the pain and secondary wound damage caused by friction on the new granulation tissue. This ASR was composed of magnetized nanoparticles (FPC Nps) and a hydrogel matrix dual‐crosslinked by phenylborate ester bonds and hydrogen bonds. Thus, the transition between the dormant and active phases of ASR was regulated by the magnetothermal effect of FPC Nps under an alternating magnetic field (AMF), which disrupted the hydrogel network. Surprisingly, magnetothermal temperatures of 43–48 °C have been proven effective in killing tumor cells and bacteria while causing minimal damage to normal tissues.^[^ ^23^, ^24^ ^]^ Therefore, in addition to controlling the phase switch, the magnetothermal properties of ASR also provided antitumor and antibacterial effects. More importantly, the actuating cores of ASR (FPC Nps) composed of dopamine and copper peroxide‐modified ferroferric oxide also served as on‐demand ROS production/scavenging nanozymes. As the pH of wounds shifted toward a weakly acidic state due to residual tumors and purulent,^[^ ^25^, ^26^ ^]^ FPC Nps acted as a POD‐like nanozyme and generated H_2_O_2_ and amplified ROS production for increased antitumor and antibacterial activity. As the wound pH fluctuated between neutral and alkaline during tissue remodeling,^[^ ^27^ ^]^ the production of H_2_O_2_ from copper peroxide was limited and the Fenton reaction was inhibited. Hence, the nanozyme did not induce ROS production, and the polyphenol groups in dopamine could scavenge ROS.

Overall, compared with traditional wound dressings, the ASR demonstrated the unique properties of painless deep wound drainage owing to its special deformability and movement. The ASR also exhibited higher biocompatibility than most previously developed magnetic soft robots due to its optimized mechanical structure, which significantly reduced the content of magnetic nanoparticles required to drive the ASR. Meanwhile, the ASR could selectively act as a ROS amplifier/scavenger to address the dynamic requirements of wound healing after tumor excision. Notably, we also explored the mechanical structure, deformation characteristics, and motion mechanisms of the ASR through force analysis and simulation with COMSOL Multiphysics^®^ software. These findings offer additional theoretical support for the development of novel hydrogel robotic dressings. Thus, bioinspired hydrogel robots that function as highly effective dressings and can painlessly drain tissue exudate, inhibit tumor recurrence, and simultaneously promote wound healing are expected to revolutionize clinical smart dressings in the future.

## 2 Results and Discussion

### 2.1 Synthesis and Characterization of ASR

**Figure 1:**
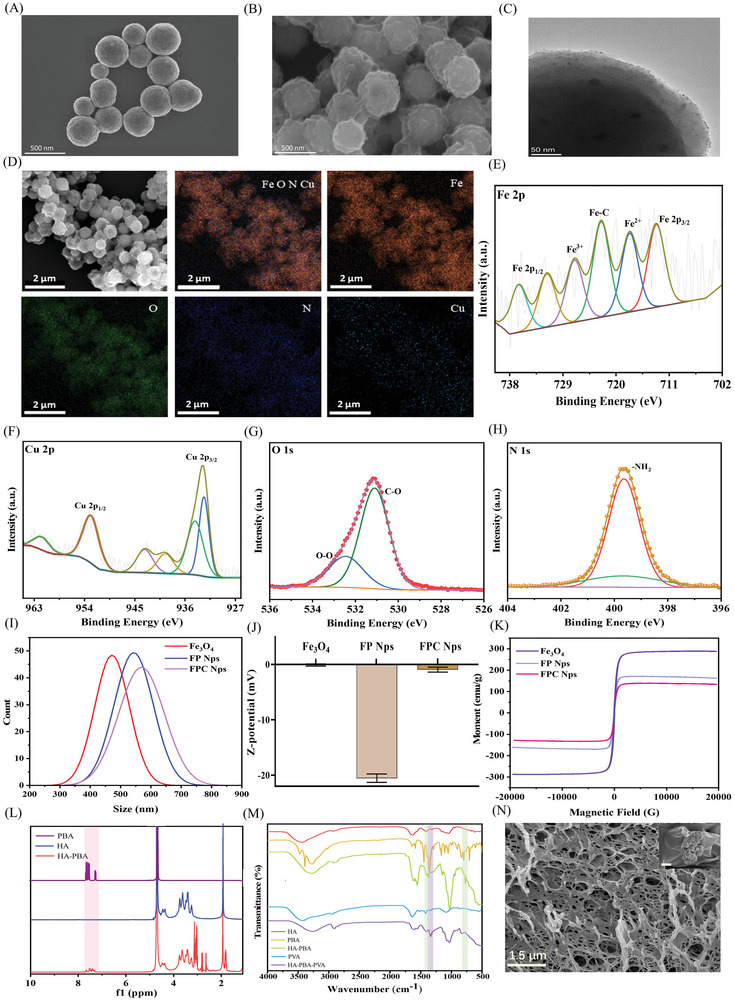
Synthesis and characterization of ASR. (A) Structure of Fe_3_O_4_ nanoparticles; scale bar: 500 nm. (B) Structure of FPC Nps characterized using scanning electron microscopy (SEM); scale bar: 500 nm. (C) Structure of FPC Nps characterized using transmission electron microscopy (TEM); scale bar: 50 nm. (D) Elemental mapping of Fe, O, N, and Cu in FPC Nps. (E‐H) High‐resolution X‐ray photoelectron spectroscopy (XPS) spectra of the Fe 2p, Cu 2p, O 1s, and N 1s signals of FPC Nps. (I) Size distribution of NPs. (J) Zeta potentials of NPs (*n* = 3). (K) Hysteresis curves of NPs. (L) ^1^H‐NMR spectra of phenylboronic acid (PBA), hyaluronic acid (HA), and HA‐PBA. (M) FT‐IR spectra of PBA, HA, HA‐PBA, polyvinyl alcohol (PVA), and HA‐PBA‐PVA. (N) Porous structure of ASR (inset: FPC Nps in the network of ASR; scale bar: 1.5 µm).

Magnet‐driven soft robots consist of magnetized (or magnetizable) particles uniformly dispersed in a soft polymer matrix.^[^ ^28^ ^]^ We first synthesized the magnetized NPs (FPC Nps). Of the magnetic components used in magnetic robots, iron oxide NPs (IONPs) have been approved for clinical use.^[^ ^29^ ^]^ Thus, Fe_3_O_4_ NPs sized approximately 450 nm were synthesized using the solvothermal method (**Figure** **1**A). Owing to their size, the Fe_3_O_4_ NPs had suitable ferromagnetism, which ensured the movement of ASR and could be degraded in vivo via phagocytosis.^[^ ^30^ ^]^ Figure 1B shows the structure of FPC Nps. The Fe_3_O_4_ NPs had a distinct coating due to dopamine aggregation on their surface. Transmission electron microscopy (TEM) analysis of FPC Nps (Figure 1C) revealed massive nanodots attached to the coating of Fe_3_O_4_ NPs. We speculated that these nanodots were ultra‐small‐sized copper peroxide particles. Therefore, elemental mapping of FPC Nps was conducted (Figure 1D). The results demonstrated that iron, oxygen, copper, and nitrogen were uniformly distributed in the FPC Nps.

Subsequently, we investigated the chemical composition of FPC Nps via XPS (Figure 1E–H; Figure S1A, Supporting Information). The peaks located at 710.01, 722.58, 712.82, 724.57, 727.4, and 732.3 eV corresponded to Fe^2+^ 2p_3/2_, Fe^2+^ 2p_1/2_, Fe^3+^ 2p_3/2_, and Fe^3+^ 2p_1/2_, respectively, and were attributed to the structure of Fe_3_O_4_. An O‐O peak appeared at 532.5 eV, and Cu^2+^ 2p_3/2_ and Cu^2+^ 2p_1/2_ showed two prominent peaks at 933.5 and 953.5 eV, respectively, confirming the existence of copper peroxide. The N 1s peaks at 399.57 eV were assigned to C‐NH_2_, indicating the presence of dopamine.

The particle size distribution and zeta potentials of FPC Nps were analyzed in Figure 1I,J. The zeta potential of FP Nps decreased due to dopamine with the negative charge of the catechol group. The particle size of FPC Nps did not change significantly after modification with copper peroxide and the zeta potential increased to 1.38 ± 0.08 mV, which was related to the positive charge of the ultra‐small size copper peroxide. The above results proved the successful synthesis of FPC Nps.

The flexible motion of ASR depends on the ferromagnetism of FPC Nps. According to previous reports, the saturation magnetization of magnetic components in soft robots typically ranges from 10 to 100 emu g^−1^.^[^ ^31^, ^32^, ^33^ ^]^ However, the saturation magnetization of FPC Nps was found to be 138.67 emu g^−1^ (Figure 1K). Hence, although the magnetism of the actuator components of ASR decreased after multiple modifications, it remained higher than reported previously.

Next, hyaluronic acid (HA) and polyvinyl alcohol (PVA) were used to synthesize the soft hydrogel matrix of the robot (Figure S1B, Supporting Information). The specific viscoelasticity of the phenylborate ester bond network was demonstrated in our previous studies,^[^ ^34^ ^]^ and we hypothesized that it would be the key to the deformation of ASR. Thus, a phenylborate ester bond network was used as the main network of ASR. In the ^1^H NMR spectra (Figure 1L), signals at 7.56, 7.58, 7.51, and 7.39 ppm corresponded to the proton‐specific signal of phenylboronic acid (PBA). In the FT‐IR spectrum (Figure 1M), the out‐of‐plane C‐H bending bands of aromatics appeared at 770 cm^−1^, the characteristic absorption bands of the benzene ring at 1550 cm^−1^, and the sharp peak of B‐O at 1340 cm^−1^. These findings implied the successful construction of the phenylborate ester bond network (HA‐PBA‐PVA). However, the overly soft phenylborate ester bond network was not elastic enough to provide the required mechanical properties for the dormant ASR. Hence, PVA crystalline domains formed by hydrogen bond interactions were introduced. The final ASR had a porous hydrogel framework, and FPC Nps were uniformly dispersed in this network (Figure 1N).

Collectively, these data demonstrated that ASR was successfully fabricated. Owing to its thoughtful design, the ASR had a main network consisting of a soft matrix with phenylborate ester bonds, which enabled its rapid deformation. PVA crystalline domains acted as the secondary network to boost the mechanical properties of the dormant ASR and confer it with suitable elasticity. Importantly, the magnetic components of ASR had stronger magnetism than previously reported soft robots, further improving its potential for deformation and motion.

### 2.2 Deformation and Controlled Manipulation of ASR

**Figure 2:**
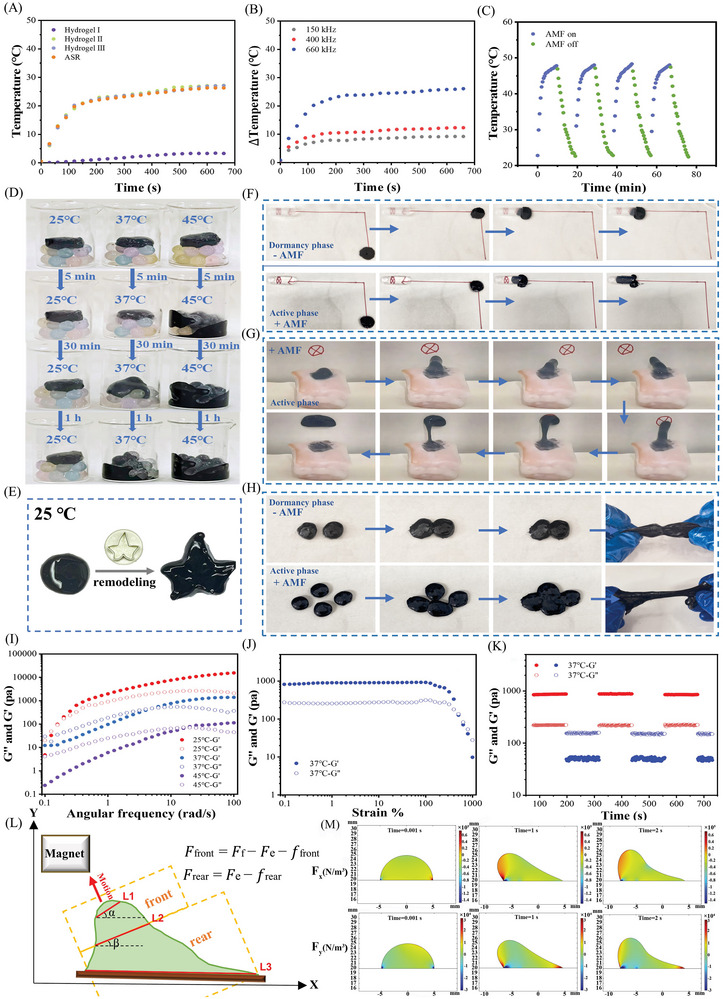
Deformation and controlled manipulation of ASR. (A) Temperature changes in hydrogels under an alternating magnetic field (alternating magnetic field, AMF; 660 kHz). (B) Temperature changes in ASR in response to different AMF power intensities (150 kHz, 400 kHz, and 660 kHz). (C) Thermal stabilities of ASR during AMF on/off cycles. (D) Mobility of ASR at different temperatures. (E) Remodeling of the dormant ASR. (F) ASR enters a small hole in the dormant and active phases. (G) Active ASR deforming and crawling out of a wound. (H) Self‐healing of ASR in the dormant and active phases. (I) Frequency scanning test of ASR. (J) Strain amplitude sweep test of ASR. (K) Alternate‐step strain sweep test of ASR. (L) Mechanical analysis of ASR. (M) Simulation of the force field distribution of ASR actuated by a 10 × 15 mm^3^ cylindrical magnet. Note: Hydrogel I: HBP hydrogel (hydrogel matrix of ASR); Hydrogel II: HBP hydrogel containing Fe_3_O_4_; Hydrogel III: HBP hydrogel containing FP Nps (Iron oxide nanoparticles coated with dopamine).

ASR was designed to exhibit solid‐like elasticity in the dormant phase to enable wound support. In contrast, it demonstrated fluid‐like mobility in the active phase, which allowed it to easily exit the wound and transport tissue exudate. The transition between the two phases was dependent on the magnetothermal properties of ASR. After 10 min of alternating magnetic field (AMF) treatment (660 kHz), the final temperature of ASR increased by 26.3 °C due to the magnetocaloric function of Fe_3_O_4_ (**Figure** **2**A). Further, dopamine and copper peroxide modification did not affect the heating properties of Fe_3_O_4_. The temperature of ASR could be controlled by adjusting AMF strength (Figure 2B), and ASR exhibited magnetothermal stability in a cyclic AMF field of 660 kHz (Figure 2C). These results showed that ASR has excellent magnetothermal properties. Figure 2D visually illustrates the effect of temperature variation on ASR. At 45 °C, ASR filled the gaps between beads within 5 min, although this process took 1 h at 37 °C and even longer at 25 °C (Figure 2D). Hence, the transition from the dormant to the active phase could be controlled by temperature changes induced by the magnetothermal effect. Moreover, the remodeling behavior of ASR at 25 °C (Figure 2E and Movie S6, Supporting Information) showed that ASR could be stuffed into the irregular shape like plasticine.

Next, we examined the phase transition, deformation, movement, and self‐healing ability of ASR. ASR could be guided to move along a predetermined orbit toward the entrance of a hole under the influence of a permanent magnet (Figure 2F; Figure S2A and Movie S1, Supporting Information). During the dormant phase, ASR was unable to enter the narrow hole and only moved around the entrance. However, after AMF exposure (660 kHz, 10 min), ASR transitioned to the active phase, entering the small hole and reaching the target owing to its deformability. Active ASR could crawl out from a tunneling wound (diameter = 10 mm, depth = 10 mm) in porcine tissue, track a moving target, and hit it (Figure 2G; Figure S2B and Movie S2, Supporting Information). Both dormant and active ASR exhibited self‐healing properties and could complete self‐healing within 30 s (Figure 2H; Figure S2C and Movie S3, Supporting Information).

Subsequently, rheological tests were performed to understand the mechanical properties of ASR. As shown in Figure 2I, the elastic modulus (G′) and viscous modulus (G′′) of ASR depended on the frequency at 25 °C. ASR appeared fluid‐like at low frequencies (0.1–0.15 rad s^−1^) and solid‐like at high frequencies (0.15–100 rad s^−1^), indicating that it could be reshaped. The intersection between G′ and G′′ occurred at a higher angular frequency position as the temperature increased, indicating that the mobility of ASR was enhanced at higher temperatures. This was attributed to the partial disruption of borate ester bonds and hydrogen bonds at higher temperatures.^[^ ^34^, ^35^ ^]^ Notably, the collapsed network could quickly recover after withstanding a maximum strain of 200% (Figure 2J,K), which confirmed the self‐healing ability of ASR, mediated by the quick re‐formation of borate ester and hydrogen bonds.

To explore the deformation and movement mechanism of ASR, the mechanical analysis of ASR was also performed (Figure 2L); further information is provided in the supporting information. Based on previous reports,^[^ ^36^ ^]^ we considered that the deformation and movement of ASR depend on three fundamental forces: the driving force generated by the magnet (*F_f_*), the cohesive force of ASR (*F_e_*), and the adhesion force (*f*_*rear*_ and *f_front_*) between ASR and the substrate (skin, muscle, etc.). Owing to the weaker magnetic force inside deep wounds, ASR could be divided into a front and rear end depending on whether it was exposed to magnetic force. Notably, the magnetic force acting on the rear of ASR was negligible. The net force at the front of ASR was as follows:

(1) $$F_{front}=F_{f}-F_{e}-f_{front}$$

The net force at the rear of ASR can be described as:

(2) $$F_{rear}=F_{e}-f_{rear}$$

Equations (1) and (2) show that the cohesive force acted both as a resistance and driving force, controlling the deformation and motion of ASR. Therefore, we orchestrated the active ASR at about 45 °C to exhibit both liquid‐like and solid‐like properties. This ensured that *F_e_* was neither too large to hinder deformation at the front end of ASR nor too small to inhibit the movement at the rear. As a result, the magnetic force driving ASR only needed to ensure movement at the front end of ASR, and its rear end could be pulled out of deep wounds owing to the cohesive force within ASR. This strategy allowed ASR to crawl out of the wound smoothly even without the strong influence of magnetic force. More importantly, as a strong magnetic was not required, ASR only contained 1.5 wt% of magnetic NPs, one order of magnitude lower than that in previously reported deformable magnetic soft robots.^[^ ^32^, ^37^, ^38^, ^39^ ^]^ This difference was critical to achieve excellent biocompatibility and biodegradability in vivo. Moreover, since the deformation size is strongly related to the adhesion force between ASR and the substrate, the maximum deformation rate of ASR at different interfaces is statistically analyzed. To simulate the deformation of ASR in a wound with exuding secretions, we investigated the maximum deformation rate of ASR at the interface of wet skin and muscle. The ASR (0.5 g, diameter = 1 cm) achieved a maximum deformation rate of up to 903.33 ± 35.59% on wet skin and 883.33 ± 46% on wet muscle. In contrast, the maximum deformation rates of ASR on the paper and plastic surfaces were significantly lower at 580 ± 30.82% and 673.33 ± 38.94%, respectively. Based on the theoretical speculations in Figure 2L, the effective adhesion force between ASR and the wet soft tissue maintains the rear of ASR in situ, while the front of ASR moves along the wet interface, resulting in lower friction compared to its mobility on plastic and paper. Consequently, ASR experiences a greater deformation rate on wet skin and muscle. These results fully demonstrated the excellent potential of ASR for drainage in wet wounds.

Simulation analysis demonstrated the forces experienced by ASR along the X and Y axes (Figure 2M; Figure S2H, Supporting Information) and further verified the deformation and displacement of ASR under AMF exposure. Briefly, the deformation and movement of active ASR depended on the balance of magnetic force, cohesive force, and adhesive force. The viscoelasticity (cohesive force) of ASR was fine‐tuned to ensure that even its rear end could climb out of the wound despite experiencing insufficient magnetic force. Further, the content of magnetic NPs added to ASR was much lower than previously reported, likely conferring ASR with improved biocompatibility and biodegradability.

Moreover, ASR exhibited significant shear‐thinning characteristics (Figure S2E, Supporting Information). In other words, when the magnetic force was relatively high, ASR could move even if the adhesive force between it and the substrate was strong because ASR could collapse and split into two parts under the influence of driving and adhesion forces. In this study, we exploited the shear‐thinning property of ASR and enhanced the interfacial force between ASR and tissues by the phenolic hydroxyl groups on dopamine.^[^ ^40^ ^]^ As expected, the portion of ASR that was tightly adhered to the wound remained in place, while the rest of the ASR absorbed tissue exudate and exited the wound, and similar trend was also observed in Figure S2G and Movie S2 (Supporting Information). Friction on new granulation tissue during dressing changes is detrimental to wound healing. However, the crawling ASR did not directly rub against the newly formed granulation tissue, reducing patient discomfort and preventing tissue damage. The ASR remaining at the wound site could protect fragile tissues during dressing changes, showed good biocompatibility, and could be degraded in vivo at the end of treatment. Thus, we can infer that ASR crawled out of the wound via “non‐direct friction” and prevented pain.

In summary, after ASR is inserted into the wound, some part of it goes deep within the wound, while some remains on the skin's surface. The portion of ASR remaining on the body surface (about 25 °C) protects the wound owing to its elasticity, while the ASR entering deep wounds (about 37 °C) self‐adapts to the irregular cavity owing to its deformability. Further, ASR crawls out of the wound after AMF exposure (about 45 °C) to remove wound exudate. However, the small portion of ASR that was firmly adhered to the wound tissues remains in situ to minimize friction. The new ASR and residual ASR merge via self‐healing to continue absorbing wound exudate and maintain therapeutic function. The residual ASR at the wound site is degraded in vivo at the end of treatment. Interestingly, due to its special viscoelasticity, ASR can climb out of deep wounds even when the external magnetic force is insufficient. Notably, the content of magnetic components in ASR is an order magnitude lower than that in previously reported magnetic soft robots, enabling ASR degradation in vivo.

### 2.3 ROS Amplification/Scavenging and O_2_ Production In Vitro

**Figure 3:**
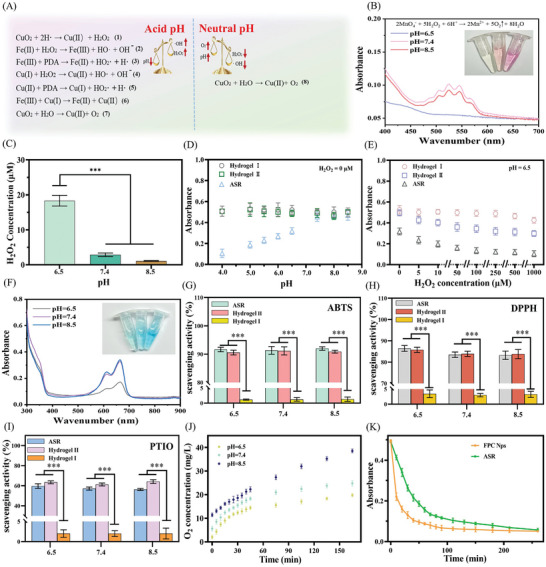
ROS amplification/scavenging and O_2_ production by ASR. (A) Mechanism of ROS amplification/scavenging. (B) KMnO_4_ colorimetric analysis of self‐produced H_2_O_2_ in ASR at pH 6.5, 7.4, and 8.5. (C) Detection of self‐produced H_2_O_2_ in ASR at pH 6.5, 7.4, and 8.5 (*n* = 3). (D) POD‐like activity of hydrogels at different pH values (*n* = 3). (E) POD‐like activity of hydrogels at different concentrations of H_2_O_2_ (*n* = 3). (F) UV–vis absorbance spectra of methylene blue (MB) solutions containing ASR at different pH values (H_2_O_2_ = 0 µm). (G–I) Free radical‐scavenging ratio of hydrogels, tested using ABTS, DPPH, and PTIO assays, respectively (*n* = 3). (J) O_2_‐generating ability of ASR (*n* = 3). (K) Time‐dependent changes in POD‐like activity (*n* = 5). Note: Hydrogel I: HBP hydrogel (hydrogel matrix of ASR) containing Fe_3_O_4_; Hydrogel II: HBP hydrogel containing FP Nps.

Wound pH exhibits dynamic changes. In wounds with residual tumors, the pH of the tumor matrix ranges from 6.0 to 7.0, whereas that of normal wound tissue fluctuates between neutral and alkaline during cell proliferation and tissue remodeling.^[^ ^26^ ^]^ Similarly, pus‐secreting wounds have a lower pH (≈pH 6.1) due to the presence of neutrophils fighting bacteria,^[^ ^25^ ^]^ but when infection and inflammation are controlled, wound pH returns to neutral/alkaline levels.^[^ ^27^ ^]^ Therefore, ASR was designed to produce or scavenge ROS on demand based on changes in the wound pH (**Figure** **3**A).

Considering that the H_2_O_2_ concentration in the wound microenvironment is insufficient to drive the Fenton reaction, we engineered ASR to self‐produce H_2_O_2_. The KMnO_4_ colorimetric assay and H_2_O_2_ quantification assay confirmed that ASR generated high levels of H_2_O_2_ under weakly acidic conditions, but not under neutral‐to‐alkaline conditions (Equation (1) in Figure 3A). The absorbance of hydrogels I and II did not change significantly at different pH values (Figure 3D; H_2_O_2_ = 0 µm), but the absorbance of ASR gradually declined with decreasing pH. This proved that ASR could selectively self‐produce H_2_O_2_ for the Fenton reaction (Equation (2) in Figure 3A), laying the foundation for the bidirectional regulation of ROS levels by ASR.

The optimal pH for iron‐based Fenton‐like reactions is typically 2.0–3.5.^[^ ^41^ ^]^ This can be ascribed to the rapid precipitation of iron ions as Fe(OH)_3_ at a neutral pH, resulting in a lower rate of Fe (III)–Fe (II) conversion and decreased electron transfer efficiency.^[^ ^42^, ^43^ ^]^ A similar trend was observed in this study (Figure 3E). Even when the concentration of H_2_O_2_ continued to increase at pH = 6.5, hydrogel I did not show an obvious Fenton reaction. Interestingly, in ASR, dopamine acted as a reducing agent to accelerate the conversion of Fe (III) to Fe (II) (Equation (3) in Figure 3A). Cu (I) reduced Fe (III) to promote the regeneration of Fe (II) (Equation (6) in Figure 3A), and Cu (II) promoted the Fenton reaction (Equations (4) and (5) in Figure 3A). Additionally, the core‐shell structure of FPC Nps enabled an efficient cascade reaction between copper peroxide and Fe (II) near the dopamine surface, with minimal effects on the magnetism of Fe_3_O_4_ and the movement of ASR. Hence, ASR generated a highly efficient Fenton reaction at pH = 6.5 and H_2_O_2_ = 0 µm (Figure 3F). That is, the cleverly designed ASR self‐supplied H_2_O_2_ and reduced iron precipitation under weakly acidic physiological conditions to accelerate Fenton oxidation and maintain a “Fe (III)‐Fe (II)‐Fe (III)” cycle, demonstrating the potential to amplify ROS around the tumor and infection site.

We then tested the ability of ASR to scavenge ROS in neutral to alkaline environments. 1,1‐diphenyl‐2‐picrylhydrazyl (DPPH) and 2, 2′‐azino‐bis(3‐ethylbenzothiazoline‐6‐sulfonic acid) (ABTS) could provide typical nitrogen free radicals; 2‐Phenyl‐4,4,5,5‐tetramethylimidazoline‐3‐oxide‐1‐oxyl (PTIO) could supply the oxygen free radicals, which were used to evaluate the ROS scavenging ability of ASR (Figure 3G–I).^[^ ^44^ ^]^ ASR exhibited excellent ROS‐scavenging ability at different pH values, which was due to the reducing phenolic hydroxyl groups on dopamine.^[^ ^45^ ^]^ We speculate that although dopamine also scavenges •OH radicals, ASR produces more •OH under acidic conditions.

Oxygen‐releasing biomaterials are increasingly being incorporated into smart wound dressings to address the problem of low oxygen supply and high oxygen demand in infected deep wounds.^[^ ^46^ ^]^ Figure 3J shows that ASR induced O_2_ and the release of O_2_ increased with increasing pH (Equations (7) and (8) in Figure 3A). This was probably because some copper peroxide nanodots were consumed to produce H_2_O_2_ under acidic conditions. Notably, the O_2_ produced by ASR was also depleted over time. Although the hydrogel matrix of ASR prolonged the duration of •OH release, this process was nearly completed within 200 min (Figure 3K). Even in previous studies, the Fenton reaction was reported to end within a few hours.^[^ ^42^, ^47^ ^]^ Thus, these O_2_ and ROS delivery systems have a short life. Nevertheless, ASR could be replaced as needed to maintain the long‐lasting release of active agents in deep wounds. Thus, it showed potential as an oxygenator and ROS generator/cleaner to meet the long‐term needs of tumor ablation, bacteriostasis, and wound healing.

In conclusion, the well‐designed ASR overcomes the limitations of the traditional iron ion Fenton reaction, which is inefficient under physiological conditions. Notably, ASR can self‐supply H_2_O_2_, amplify ROS in weakly acidic physiological conditions (residual tumors and infected tissues), and produce oxygen and scavenge ROS in neutral‐to‐weakly alkaline environments (pH of remodeling tissue). Thus, ASR may demonstrate high antitumor and antibacterial efficacy while promoting tissue regeneration.

### 2.4 Adhesive Properties of ASR in Different Phases

Traditional dressings are often held in place by additional fixation methods and thus fall off easily. Hence, dressings with wet tissue adhesion have attracted widespread attention.^[^ ^48^ ^]^ However, these dressings are often difficult to remove from deep wounds. To address these challenges, ASR was designed to exhibit strong wet tissue adhesion properties in the dormant phase, enabling easy clinical application, and poor adhesion properties in the active phase, which allowed it to flexibly climb out of wounds.

**Figure 4:**
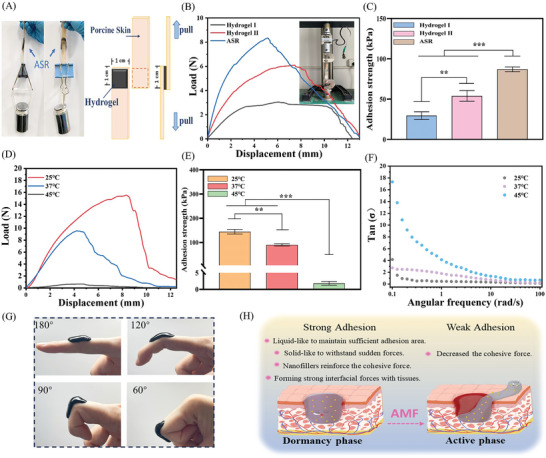
Adhesion capacity of ASR. (A) Adhesion behavior of ASR on porcine skin. (B) Load‐displacement curves of hydrogels at 37 °C. (C) Adhesion strength of hydrogels at 37 °C (*n* = 3). (D) Load‐displacement curves of ASR at different temperatures. (E) Adhesion strength of ASR at different temperatures (*n* = 3). (F) Damping factor of ASR at different temperatures. (G) Adhesion of ASR to moving joints. (H) Adhesion and low‐adhesion mechanisms of ASR. Note: Hydrogel I: HBP hydrogel (hydrogel matrix of ASR); Hydrogel II: HBP hydrogel containing Fe_3_O_4_.

To visualize the adhesion properties of ASR in the dormant phase, we glued two pieces of pig skin using the dormant ASR at 25 °C (**Figure** **4**A). The dormant ASR could lift heavy objects weighing 500 g (adhesion area = 1.0 × 1.0 cm), indicating its high adhesion strength. Subsequently, we quantified its adhesion strength and explored the potential factors affecting adhesion using lap shear tests. The adhesion strength of hydrogel I was 29.56 ± 1.92 kPa at 37 °C (Figure 4B,C), higher than that of some reported hydrogels.^[^ ^8^, ^40^, ^49^ ^]^ Hydrogel I had both liquid‐like and solid‐like properties. Thus, we speculated that it could self‐adapt to the uneven surface of the skin to acquire a sufficient adhesion area, while withstanding sudden forces due to its solid‐like elasticity, thus achieving a strong adhesion effect. Meanwhile, according to the filler reinforcement mechanism,^[^ ^50^, ^51^ ^]^ Fe_3_O_4_ NPs reinforced the mechanical properties (usually equivalent to the cohesive force) of hydrogel II, resulting in an adhesive strength of 53.91 ± 3.83 kPa. These mechanisms contributed to the adhesion strength of ASR, and the phenolic hydroxyl groups on the surface of FPC Nps further strengthened the interfacial force between ASR and wet tissue.

Next, we investigated the adhesion properties of ASR in the active phase. The adhesion strength of ASR reached its lowest value during the switch from the dormant phase (25 °C) to the active phase (45 °C) (Figure 4D,E). The damping factor tan (δ) (G″/G′) illustrated the relative influence of ASR viscosity and elastic behavior.^[^ ^52^ ^]^ The mobility of ASR increased with the temperature (Figure 4F), implying that active ASR had poorer cohesion properties than dormant ASR, which was the critical reason for the decrease in its adhesion strength. Figure 4G elucidates how dormant ASR adhered to a moving joint. Finally, the strong adhesion of dormant ASR and weak adhesion of active ASR are summarized in Figure 4H.

### 2.5 In Vitro Anti‐Bacterial Performance of ASR

**Figure 5:**
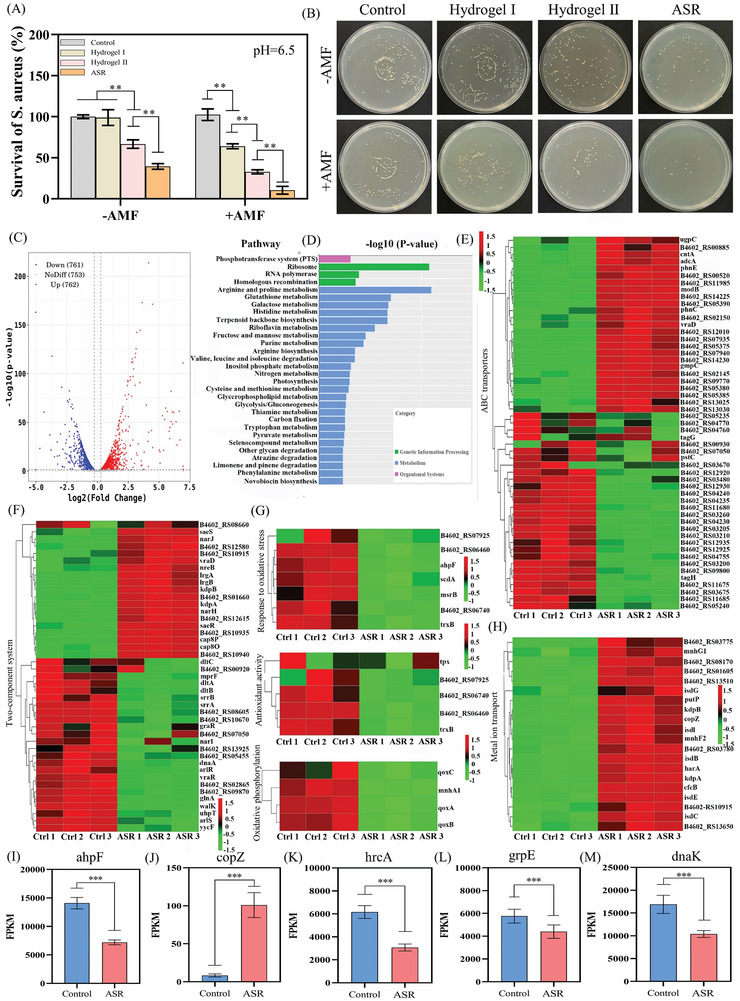
Anti‐bacterial performance of ASR. (A) Anti‐bacterial performance of hydrogels tested using direct contact methods (*n* = 3). (B) Bacterial colonies of *S. aureus*. (C) Volcano plot analyses of differentially expressed genes (DEGs) (*n* = 5). (D) KEGG analyses of DEGs (*n* = 5). (E) Heatmap of genes associated with ABC transporters (*n* = 5). (F) Heatmap of genes associated with the two‐component system (*n* = 5). (G) Heatmap of genes associated with oxidative stress (*n* = 5). (H) Heatmap of genes associated with metal ion transport (*n* = 5). (I–M) Fragments per kilobase million (FPKM) of (I) *ahpF*, (J) *copZ*, (K) *hrcA*, (L) *grpE*, and (M) *dnaK* in *S. aureus* (*n* = 5). Note: Hydrogel I: HBP hydrogel (hydrogel matrix of ASR) containing Fe_3_O_4_ Nps; Hydrogel II: HBP hydrogel containing FP Nps.

*Staphylococcus aureus* (*S. aureus*) is among the key strains causing wound infections.^[^ ^53^ ^]^ Thus, it was used as a representative strain for evaluating the antibacterial activity of ASR in vitro under weakly acidic conditions (**Figure** **5**A,B). The survival rate of *S. aureus* in the hydrogel II group was lower than that in the control and hydrogel I groups, and this value decreased further after AMF exposure. Notably, ASR+AMF achieved greater bacterial lethality than hydrogel II+AMF. These results suggested FP Nps, FPC Nps, and AMF may all contribute to antibacterial effects.

Next, the antibacterial mechanism of ASR was explored via whole‐transcriptome RNA sequencing. As shown in Figure 5C, 1523 differentially expressed genes (DEGs) were identified between bacteria from the ASR group versus the control group. These DEGs were enriched in KEGG pathways associated with “genetic information processing,” “biological systems,” and “metabolism.” Among them, “ribosome” and “Arginine and proline metabolism” were the most altered pathways (Figure 5D). In harsh environments, active *S. aureus* ribosomes maintain a fast translation rate to synthesize stress‐responsive proteins and ensure survival.^[^ ^54^ ^]^ Of the 58 genes associated with ribosome function in ASR‐treated *S. aureus*, 46 showed altered expression (Figure S3A, Supporting Information), suggesting that ASR induces a stress‐responsive state in *S. aureus*.

Arginine can be metabolically exchanged with other amino acids (such as proline) and synthesize nitric oxide, polyamines, and urea cycle metabolites. *S. aureus* can use the metabolic intermediates produced via the degradation of these amino acids as carbon sources.^[^ ^55^ ^]^ The 10 DEGs related to arginine and proline metabolism in ASR‐treated *S. aureus* showed low transcription levels (Figure S3B, Supporting Information), reducing the production of endogenous carbon and nitrogen sources in bacteria and ultimately hindering bacterial growth.

Proline is an essential amino acid involved in osmotic regulation and the maintenance of membrane integrity.^[^ ^56^ ^]^ Hence, these results also implied that ASR disrupted the cell membranes of *S. aureus*. The ABC transporter family is involved in active transport against the concentration gradient.^[^ ^57^ ^]^ An obvious difference in genes encoding ABC transporters was observed after ASR treatment (Figure 5E), further proving that ASR caused membrane damage. Previous reports show that dopamine can denature proteins in bacterial cell membranes,^[^ ^58^ ^]^ which may be one of the causes of the membrane damage observed in our study.

The two‐component signal transduction system (TCS) is the primary stimulus‐response coupling mechanism in prokaryotes and is closely related to bacterial metabolism and virulence.^[^ ^59^ ^]^ Among the genes involved in the TCS (Figure 5F), most DEGs encoding pathogenesis‐related toxins showed decreased expression. Genes related to oxidative stress in bacteria of the ASR group were downregulated (Figure 5G), including the *ahpF* gene encoding an alkyl hydroperoxide reductase (Figure 5I).^[^ ^60^ ^]^ This reductase can eliminate low concentrations of H_2_O_2_ from cells. Hence, the findings indicated that the higher amounts of ROS produced by ASR overwhelmed *S. aureus* cellular defenses and inhibited intracellular antioxidant activity.

Several genes related to metal ion transport and iron homeostasis also displayed altered expression (Figure 5H; Figure S3C,D, Supporting Information). Genes related to copper ion binding, especially *copZ* (involved in the cytoplasmic buffering and clearance of copper ions), were upregulated (Figure 5J).^[^ ^61^ ^]^ These results suggested that large amounts of iron and copper ions were released from ASR and transported intracellularly, disrupting bacterial iron homeostasis and exacerbating intracellular ROS production.

*S. aureus* was stimulated to synthesize heat‐shock proteins for defense in response to high temperatures. The HrcA regulon (*hrcA‐grpE‐dnaK‐dnaJ* and *groESL*) is among the most widely distributed negative heat‐shock regulators in bacteria.^[^ ^62^ ^]^ Genes encoding the HrcA regulon were downregulated in the ASR group, proving that *S. aureus* was under heat stress due to the magnetothermal effect of ASR (Figure 5K–M).

In summary, the significant antibacterial effect of ASR under AMF exposure and the weakly acidic conditions caused by bacterial colonization results from the synergistic effect of ROS amplification, dopamine interference, metal ion release, and magnetocaloric effects. These effects destroy bacterial cell membranes, disrupt bacterial metabolism, cause intracellular redox and iron homeostasis imbalances, and hinder ribosomal function in bacteria.

### 2.6 Antitumor, ROS Amplification/Scavenging, and O_2_ Production In Vitro

**Figure 6:**
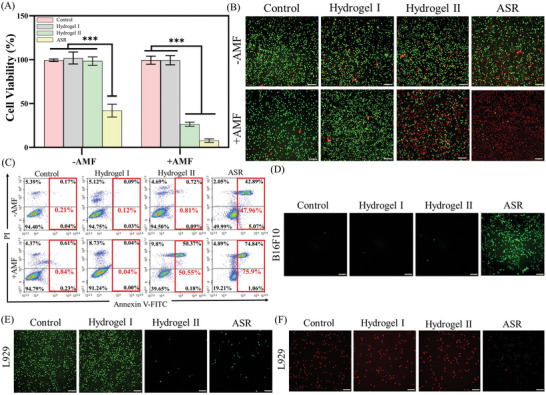
Antitumor, ROS amplification/scavenging, and O_2_ production in vitro. (A) B16F10 cell viability after treatment (pH = 6.5) (*n* = 3). (B) Live/dead staining of B16F10 cells after treatment (pH = 6.5). (C) Flow cytometry analysis of B16F10 cell apoptosis after treatment (pH = 6.5), and quantified results of apoptotic cells are shown in the red box. (D) ROS amplification in B16F10 cells (pH = 6.5). (E) ROS scavenging in L929 cells (pH = 7.4). (F) O_2_ production in anoxic L929 cells (pH = 7.4). Scale bar: 100 µm. Note: Hydrogel I: HBP hydrogel (hydrogel matrix of ASR); Hydrogel II: HBP hydrogel containing FP Nps.

The potential antitumor and pro‐healing abilities of ASR were evaluated in vitro. As shown in **Figures** **6**A,B and S4A (Supporting Information), ASR exhibited no cytotoxicity against normal fibroblasts (L929 cells) under neutral conditions (pH of normal, remodeling wound tissue). However, the viability of melanoma cells (B16F10 cells) was reduced to 41.94 ± 4.26% after treatment with ASR in a weakly acidic environment (pH of the tumor microenvironment). Additionally, treatment with ASR+AMF further reduced the viability of B16F10 cells to 7.66 ± 1.17%. This tumoricidal ability of ASR was mediated by tumor cell apoptosis (Figure 6C). These above results demonstrated that ASR could selectively induce cytotoxicity based on pH differences between tumors and normal wound tissues and produce synergistic antitumor effects via magnetocaloric therapy.

Next, we investigated whether ASR‐mediated tumor cell death is caused by ROS amplification under weakly acidic conditions. Green fluorescence was stronger in B16F10 cells of the ASR group (Figure 6D; Figure S4B, Supporting Information), indicating that ASR induced high •OH production, increasing intracellular ROS in B16F10 cells under weakly acidic conditions. Meanwhile, ASR produced the opposite effect in an L929 cell model with high ROS expression (induced by 2,2′‐AAPH^[^ ^63^ ^]^) at pH 7.4 (Figure 6E; Figure S4C, Supporting Information), dramatically reducing ROS levels. This was because the catechol groups in dopamine scavenged ROS and the Fenton reaction was blocked under neutral conditions. Hence, ASR could selectively switch its ROS scavenging/amplifying function according to pH changes in the wound microenvironment, addressing the conflicting need for ROS reductions for wound healing and ROS elevations for tumoricidal effects.

Wounds are often hypoxic, and severe hypoxia hinders wound healing and increases the risk of infection.^[^ ^64^ ^]^ To confirm the oxygen‐generating property of ASR during tissue remodeling, a fibroblast hypoxia model was established at neutral pH (Figure 6F; Figure S4D, Supporting Information). Hypoxia (red fluorescence) was found to be alleviated in the cells in the ASR group.

Previous studies show that certain NPs with targeting ability can selectively increase intracellular ROS levels in tumor cells without inducing ROS lethality in normal cells.^[^ ^65^ ^]^ However, targeting residual tumor cells at the wound site after tumor resection is challenging. Most NPs are inevitably absorbed by normal tissues, creating an additional metabolic burden. In comparison, ASR can switch its extracellular ROS production/scavenging activity based on the pH of the microenvironment and exit the wound to reduce metabolic stress in vivo. During the tug‐of‐war between targeted residual tumor clearance and wound healing, this bidirectional ROS regulation dynamically prioritizes the most urgent problems. In the absence of tumor activity (wound pH fluctuating between neutral and weakly alkaline), ASR focuses on scavenging ROS to promote wound healing, leaving the elimination of the few residual tumor cells to magnetothermal therapy and preventing the side effects of excessive antitumor therapy. In the presence of tumor activity (local wound acidification), ASR concentrates on killing tumors by amplifying ROS to enable synergistic anti‐cancer therapy. Additionally, ASR attenuates cellular hypoxia to promote wound healing.

### 2.7 Healing of Infected Full‐Thickness Skin Defect Wounds In Vivo

**Figure 7:**
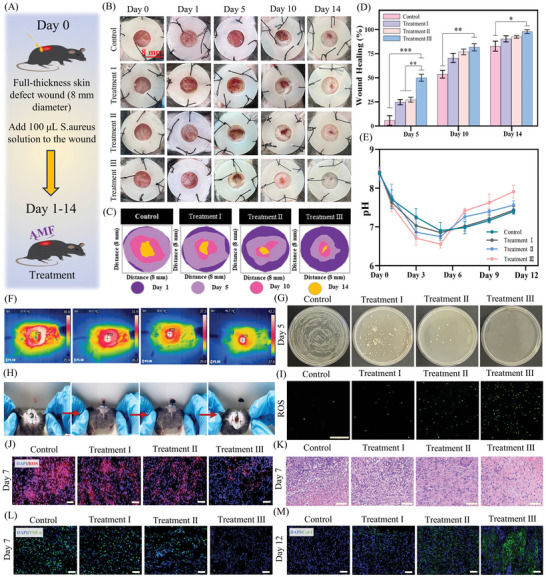
Healing of infected full‐thickness skin defect wounds (*n* = 6 per group). (A) Schematic representation of wound models and treatment strategies. (B) Images of wounds at days 0, 1, 5, 10, and 14. (C) Analysis of progress in wound healing. (D) Wound‐healing rate on days 5, 10, and 14 (*n* = 3). (E) pH of the wound during healing (*n* = 3). (F) Magnetic heat therapy with ASR. (G) Culture of bacteria collected from the wound site on day 5. (H) Images of active ASR crawling out of wounds. (I) ROS levels in bacteria collected from the wound site on day 5; scale bar: 100 µm. (J) ROS levels in wounds on day 7; scale bar: 40 µm. (K) H&E staining of wound tissue; scale bar: 60 µm. (L) Immunofluorescence staining for TNF‐α at the wound site on day 7; scale bar: 40 µm. (M) Immunofluorescence staining for collagen I (Col‐I) at the wound site on day 12; scale bar: 40 µm. Note: the grouping scheme is provided in the Experimental section.

The motion and potential biological applications of deformable magnetic soft robots have recently been simulated in vitro.^[^ ^66^, ^67^, ^68^ ^]^ However, only a handful of studies have examined the in vivo application of soft robots for addressing clinical challenges.^[^ ^69^ ^]^ To verify the antibacterial and wound‐healing effects of ASR in vivo, infected full‐thickness skin defect models were established. **Figure** **7**A illustrates the process of wound treatment, and Figure 7B–D shows the efficacy of various treatment strategies in promoting wound healing. On days 5 and 10, the wounds in treatment groups I‐III showed a smaller unclosed wound area than those in the control group, with treatment III demonstrating the best therapeutic effect.

To elucidate the therapeutic mechanism of ASR, we investigated its magnetothermal action and movement in vivo (Figure 7F). Initially, the temperature of ASR was 27 °C; at this temperature, ASR provided sufficient elasticity for wound protection. Following AMF exposure, the temperature of ASR increased from 27 to 46.7 °C, demonstrating its excellent magnetothermal therapeutic potential. After the completion of magnetothermal treatment (≈45 °C for 10 min), ASR showed deformability and could climb out of the wound under the guidance of a static magnetic field (Figure 7H and Movie S4, Supporting Information). However, full‐thickness skin defects represent relatively superficial damage, and these wounds are not sufficient to verify the clinical feasibility of ASR in deep wounds. Thus, the advantages of ASR in deep wound treatment are discussed in additional detail in section 2.9.

The on‐demand production/scavenging of ROS by ASR is dependent on the dynamic changes in wound pH during the healing process. Hence, we measured wound pH values during healing in this study (Figure 7E). Consistent with previous reports,^[^ ^25^ ^]^ the wound microenvironment was weakly acidic in the early stage of healing (3–5 days) but maintained a neutral‐to‐alkaline pH during the mid‐to‐late stages of wound healing (7–14 days). Subsequently, we explored whether ASR can selectively scavenge/amplify ROS depending on the wound pH in vivo. On day 3 (weakly acidic wound microenvironment), ROS accumulation was detected in the bacteria at the wound site (Figure 7G–I; Figure S5E, Supporting Information). Re‐cultured bacteria from treatment groups I–III exhibited higher ROS accumulation than those from the control group, and their bacterial counts were lower. ASR with magnetothermal treatment (treatment II) increased ROS accumulation in bacteria, accelerating bacterial death. Notably, treatment group III showed the strongest antibacterial efficacy, indicating that the daily replacement of ASR enabled a continuous •OH supply for long‐lasting antibacterial effects. Additionally, ASR reduced the proliferation of other bacteria within the wound, exhibiting broad‐spectrum antibacterial action. Thus, ASR could amplify intracellular ROS in bacteria by responding to the low pH of the wound environment, thereby inhibiting bacterial proliferation. The greatest antibacterial effect was achieved through combined magnetothermal therapy and daily ASR replacement.

The process of wound healing involves four phases—blood coagulation, inflammation, cell proliferation, and wound remodeling.^[^ ^8^ ^]^ It is important to reduce ROS levels during the last two stages. Hence, to verify the ability of ASR to scavenge ROS and accelerate wound healing, we assessed ROS levels in wounds on day 7 (neutral to alkaline conditions) (Figure 7J; Figure S5A,F, Supporting Information). Treatment groups I–III showed lower ROS levels in wounds than the control group and the lowest ROS levels were detected in the treatment III group. At the same time, we evaluate the H&E staining of these wounds (Figure 7K; Figure S5B, Supporting Information). The presence of inflammatory cells infiltrating the granulation tissue in both the control and treatment I groups. However, wounds in treatment groups II and III contained fewer inflammatory cells and exhibited epithelial layer formation, revealing that they had crossed the inflammatory phase and were undergoing tissue matrix remodeling. Treatment III provided the thickest epidermis and the most collagen fiber deposition, showcasing the strongest recovery effect. A similar trend was observed for tumor necrosis factor (TNF‐α), a pivotal indicator of the inflammatory response (Figure 7L; Figure S5C,G, Supporting Information). That is, TNF‐α levels were the lowest in treatment group III. Inflammation and ROS are interdependent. ROS production increases wound inflammation, which in turn exacerbates ROS levels.^[^ ^70^ ^]^ Excessive ROS levels and inflammation delay wound healing. Our findings indicated that ASR scavenges ROS in a neutral‐to‐alkaline wound microenvironment during the cellular proliferation and wound remodeling phases and shortens the inflammatory period by controlling infection. ASR breaks the vicious cycle of inflammation and ROS to efficiently promote wound healing. Importantly, the regular on‐demand replacement of ASR and its combination with magnetothermal therapy can provide superior ROS scavenging and anti‐inflammatory effects owing to enhanced antibacterial activity and the timely removal of tissue exudate.

Collagen is the primary supporting structure of skin tissue.^[^ ^71^ ^]^ The expression of collagen isoform type I (Col‐I) was higher in treatment group III than in the other groups (Figure 7M; Figure S5D,H, Supporting Information), signifying the excellent effect of treatment III on collagen production and the rapid reconstruction of functional skin tissue.

In summary, ASR adopts both the dormant phase and the active phase during the treatment of infected full‐thickness skin defects. Dormant ASR protects wounds owing to its mechanical properties and can also bi‐directionally regulate ROS in tandem with the complex wound‐healing process. In the presence of wound infections (weakly acidic environment), ASR can induce a ROS storm to eliminate bacteria, shortening the inflammatory period. Meanwhile, during cellular proliferation and wound remodeling (neutral‐to‐alkaline conditions), ASR can serve as a ROS scavenger to promote the reconstruction of skin tissue and prevent excessive inflammation. Interestingly, following AMF exposure, ASR switches from the dormant phase to the active phase owing to the magnetothermal effect, quickly crawling out of the wound to timely remove tissue exudate and dead bacteria. This further accelerates wound healing, and the antibacterial properties of ASR are further strengthened by the magnetothermal effect.

### 2.8 Healing of Wounds After Tumor Resection In Vivo

**Figure 8:**
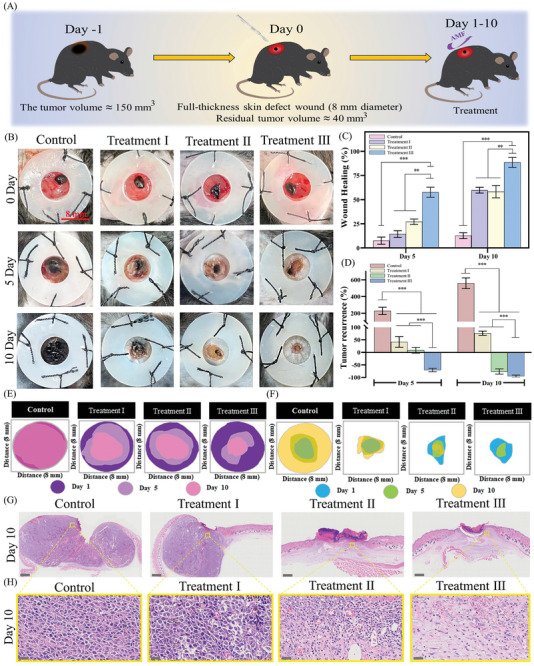
Wound healing after tumor resection (*n* = 6 per group). (A) Schematic illustration of wound models and treatment strategies. (B) Images of wounds at days 0, 5, and 10. (C) Wound‐healing rate at days 5 and 10 (*n* = 3). (D) Tumor recurrence rate at days 5 and 10 (*n* = 3). (E) Analysis of wound healing trends. (F) Analysis of tumor recurrence trends. (G,H) H&E staining of wound tissues on day 10; scale bar: 1000 µm (control and treatment I groups) and 600 µm (treatment II and III groups) (G), with tissue indicated in yellow boxes magnified in (H); scale bar: 30 µm. Note: the grouping scheme is provided in the Experimental section.

An incomplete tumor resection model was established to evaluate the ability of ASR to inhibit the local recurrence of residual tumors and promote wound healing in vivo (**Figure** **8**A; Figure S6A, Supporting Information). Examination of the time‐dependent pH changes in wound and tumor tissues (Figure S6B,C, Supporting Information) revealed that tumor tissues remained weakly acidic for 10 days, while wound tissues tended to be weakly acidic to neutral/alkaline. Therefore, the wound microenvironment containing residual tumors could also trigger the on‐demand ROS amplification/scavenging system of ASR. Next, we recorded the healing process of wounds treated using different strategies. On day 10, the wounds in the control group had hardly healed, and the residual tumor tissue gradually covered the damage. In contrast, the wounds in treatment group III showed almost no visible residual melanoma, and their wound closure rate was the highest (Figure 8B–F). This demonstrated the excellent effect of treatment III in inhibiting tumor recurrence and promoting wound healing.

Upon histologic evaluation (Figure 8G,H), wounds in the control and treatment I groups showed masses of melanoma cells, indicating that the antitumor effect of treatment I was limited. No visible accumulation of melanoma cells was detected in treatment groups II and III, but these groups showed a large amount of neovascularization, indicating that the reconstruction of functional skin tissue had begun. Treatment group III had newer epithelium and less inflammatory cell infiltration than treatment group II. In summary, ASR combined with magnetothermal therapy enabled the effective inhibition of local tumor recurrence. Hence, timely ASR replacement was beneficial for maintaining long‐term •OH release, thereby improving antitumor effects and accelerating wound healing.

### 2.9 Healing of Infected Sinus Tract Wounds In Vivo

**Figure 9:**
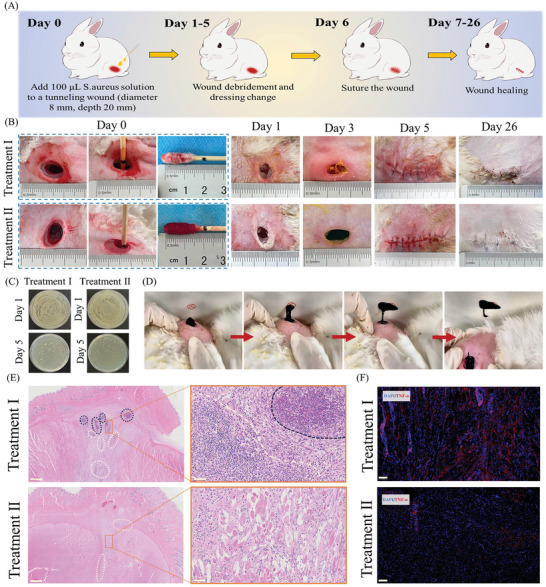
Healing of infected sinus tract wounds (*n* = 4 per group). (A) Schematic illustration of wound models and treatment strategies. (B) Images of wounds. (C) Culture of bacteria collected from the wound site on days 1 and 5. (D) Images of active ASR crawling out of wounds. (E) H&E staining of wound tissues on day 26; images on the right side show the magnified tissue indicated by the orange box in the images on the left side. The purulent area is indicated in dark purple circles and inflammatory infiltration in white circles. Scale bar: 1000 and 50 µm. For consistency and aesthetics of the layout, the enlarged cell section of the Treatment I group was rotated by 90 degrees clockwise. (F) Immunofluorescence staining for TNF‐α at the wound site on day 26; scale bar: 100 µm. Note: the grouping scheme was provided in the Experimental section.

Excessive wound exudate caused by fat liquefaction and infection is a common clinical problem in postoperative wounds, especially after large surgical incisions, such as those required for resecting malignant soft tissue tumors. These wounds typically require packing dressings or negative pressure suction devices for exudate drainage. However, these strategies increase patient distress, especially during dressing changes for irregular deep wounds. Dressings for postoperative wounds after tumor resection have rarely focused on deep wound drainage. To address this clinical requirement, we developed an animal model of an infected sinus tract wound requiring drainage and verified the performance of ASR in deeper and more extreme wounds. As shown in **Figure** **9**A, a tunneling wound (8 mm diameter, 20 mm depth) was established. Then, an *S. aureus* suspension was added to the wound for 24 h. Purulent secretions containing masses of bacteria were subsequently detected in the wounds (Figure 9C), demonstrating the successful establishment of the animal model.

Next, we confirmed the ability of ASR to painlessly remove wound secretions from deep wounds. The ASR crawling out of wounds post‐treatment was heavier than the original ASR, indicating that it had transported tissue exudate out of the wound (Figure S7B, Supporting Information). This was due to the excellent swelling properties of the hydrogel skeleton of ASR (Figure S7C, Supporting Information). In clinical settings, several patients prefer negative pressure suction devices to remove exudate from infected sinus tract wounds as this is less painful than traditional dressing changes. However, inadvertent movement during suction can alter the device's suction angle and pressure. Hence, medical staff must regularly check the suction conditions and promptly address issues such as air leakage, the inability to aspirate pus, and thrombus, which increases their workload. Figure 9D and Movie S5 (Supporting Information) demonstrate how ASR crawls out of deep wounds. Even though the rabbit moved several times during the dressing change in this study, ASR still crawled to the designated position and hit its target, demonstrating its convenience for clinical application. Moreover, at the end of the dressing change, the small portion of ASR that was firmly adhered to the wound tissue remained in situ (Movie S5, Supporting Information). This prevented direct friction on the new granulation tissue, preventing secondary wound damage and reducing pain. Hence, the rabbit did not struggle intensely during the replacement of ASR even though it was not anesthetized. These results provide compelling evidence showing that ASR has exceptional deformation and movement capabilities and can rapidly drain wound exudate and reduce patient pain during dressing changes.

Subsequently, we explored the effects of ASR on promoting the healing of infected sinus tract wounds (Figure 9B). On day 5, unlike wounds in the untreated group, wounds in treatment groups I and II no longer showed excessive wound exudate and met the criteria for suturing. The bacterial counts in the wound exudate in treatment groups I and II were also reduced (Figure 9C), showing that both treatment methods inhibited bacterial proliferation in deep wounds. Figure S7E (Supporting Information) illustrates the suturing procedure, in which residual ASR was sutured into the wound and allowed to degrade. Twenty‐six days after suturing, wounds were analyzed histologically (Figure 9E). To clarify the location of the selected lesion, we have labeled the area of selected tissue within the orange box in the left panel, which is larger than the actual area of the right cell section, while still including the entirety of the right cell section. Secondary purulent secretions and extensive inflammatory cell infiltration were observed in treatment group I. However, treatment group II showed no apparent suppurative area and low inflammatory cell infiltration, and some surviving striated muscle cells remained. TNF‐α levels were lower in wounds after treatment II (Figure 9F; Figure S7D, Supporting Information), suggesting that ASR had excellent anti‐inflammatory capabilities. These findings demonstrated that ASR was more effective in promoting the healing of infected sinus tract wounds than standard clinical procedures. This was because ASR could self‐adapt to irregular wounds, which allowed it to fully absorb tissue exudate and provide enhanced protection against bacterial invasion, thus reducing the risk of secondary wound infection. Further, tissue exudate removal by ASR did not cause wound damage, and the residual ASR sutured into the wound tissue showed prolonged antibacterial and anti‐inflammatory activity.

No obvious residual ASR was detected in the wound at 26 days after suturing, suggesting that most of the ASR had been degraded. This was likely due to phagocytosis by multinucleated giant cells (Figure S7F, Supporting Information). H&E staining (Figure S7G,H, Supporting Information) confirmed that ASR did not significantly affect the structure and function of the liver and kidney in rabbits. Given that these organs are the primary contributors to NPs excretion,^[^ ^33^ ^]^ these findings proved that ASR was relatively safe in vivo.

Taken together, ASR showed special deformation and movement advantages during the treatment of infected sinus wounds. It showed self‐adaptation to irregularly shaped wounds but could quickly crawl out of them to remove wound exudate. Interestingly, this process appeared almost painless and was not affected by the subject's posture and movement, indicating the convenience of ASR for clinical application. Furthermore, ASR exhibited better pro‐healing capabilities than standard clinical care procedures due to rapid pus removal, excellent antibacterial and anti‐inflammatory effects, and superior barrier formation. Notably, the ASR remaining inside the wound showed favorable bio‐degradability and also served as a long‐lasting antibacterial and anti‐inflammatory agent, further accelerating tissue regeneration.

## 3 Conclusion

Preventing tumor recurrence and deep wound infection are major challenges after melanoma excision. Additionally, dressing changes and the drainage of deep postoperative wounds can easily cause secondary wound damage and increase patient pain. To solve these problems, a soft robot with switchable dormant and active phases was developed herein. The transition between the dormant and active phases was dependent on the magnetothermal effect of ASR, which also provided synergistic antitumor and bacteriostatic effects. The dormant ASR supported the wounds while acting as an on‐demand ROS producer/scavenger to meet dynamic ROS needs at the wound site. It reduced ROS during tissue regeneration but increased ROS during the antitumor and antibacterial phase. Meanwhile, the active ASR removed absorbed wound exudate by crawling out of the wound, alleviating patient pain by only inducing “non‐direct friction” during the crawling process. An investigation of the deformation and motion characteristics of the ASR revealed that the hydrogel robot could smoothly crawl out of wounds even without the influence of a strong magnetic force, as long as the balance of the magnetic force, the cohesive force of the robot, and the adhesive force between the robot and tissues was maintained. Therefore, only 1.5 wt% of magnetic NPs were required to drive the ASR. This value was one order of magnitude lower than that used in previously reported magnetic soft robots, conferring the ASR with excellent biocompatibility and biodegradability in vivo. In rabbit experiments in vivo, the ASR crawled out of sinus tract wounds (8 mm in diameter, 20 mm in depth) within 50 s. During this period, the rabbits lay comfortably on their sides, showing little struggle, which demonstrated the painless drainage properties of the ASR. Importantly, the ASR could promote the healing of infected wounds and achieve 100% residual tumor inhibition, highlighting its potential as a dressing for wounds after tumor excision. Beyond tumor excision wounds, several deeper wounds – such as anal fistulas, preauricular fistulas, mastitis abscesses, perianal abscesses, and diabetic sinus tract wounds – are also challenging to manage, and urgent wound drainage solutions are required for such cases. In the future, we aim to focus on improving the actuation capability of the ASR and enhancing its performance in the management of more complex wounds.

## 4 Experimental Section

### Materials

Hyaluronic acid (HA, M_W_ = 100–200 kDa), 3‐aminophenylboronic acid (PBA), Poly(vinyl alcohol) (PVA, *M_W_* = 205 kDa) were supplied by Shanghai Macklin Biochemical Co., Ltd. (China). Iron chloride hexahydrate (FeCl_3_·6H_2_O), cupric chloride dihydrate (CuCl_2_·2H_2_O), and dopamine hydrochloride were purchased from Shanghai Aladdin Bio‐Chem Technology Co., Ltd (China).

### Synthesis of ASR

1) Synthesis of HA‐PBA: cross‐linked 3‐aminophenylboronic acid and HA with the EDC/NHS method. 2) Synthesis of FPC Nps: Fe_3_O_4_ nanoparticles were synthesized by the thermal decomposition method and self‐polymerized dopamine on the Fe_3_O_4_ surface to get FP NPs. Finally, copper peroxide was formed in situ on the dopamine. 3) Synthesis of ASR: HA‐PBA (2 wt%) and PVA (10 wt%) were mixed in mass ratio of 1:2, while 1.5 wt% FPC Nps were added to obtain a single network hydrogel. Next, the cycle freeze‐thawed twice (freeze at −20 °C for 3 h and thawed at 25 °C for 5 h) to get ASR. The hydrogel matrix of ASR (denoted as HBP hydrogel) was prepared by the same method as above, and without adding FPC Nps.

### Characterization

The hysteresis loops of nanoparticles were determined. The chemical structure of components, micromorphology, rheological characteristics, and equilibrium swelling ratio (ESR) were also measured. More experimental details are provided in the supporting information.

### Drive, Simulation, and Computation

Sintered rubidium iron boron magnet (N48) for driving ASR motion. The red target in movies and images was located at the center of the magnet, and the peak of surface magnetic field strength was about 800 mT. Commercial finite element software COMSOL Multiphysics was used to simulate the shape‐changing and movement of ASR.

### Animal Wound Healing Test

All animal experiments of this study followed the guidelines set of experimental animal management and welfare ethics of the University of Electronic Science and Technology of China (accreditation number: 1061423022725123). Mouse models of infected full‐thickness excisional wounds and full‐thickness excisional wounds with incompletely resected tumors and rabbit models of infected sinus tract wounds were established. Mice were randomly divided into four groups (*n* = 6 per group), as follows: control: wounds dressed with gauze; treatment I: wounds dressed with 0.5 g ASR; treatment II: wounds dressed with 0.5 g ASR + external AMF applied to maintain wound temperature around 45 °C (10 min) every 48 h; and treatment III: same as treatment II, except the ASR was also changed every 48 h. The rabbits were divided into two groups (*n* = 4 per group) as follows: Treatment I: wounds managed based on standard clinical procedure (daily debridement with iodized cotton swabs and gauze replacement to drain pus). Treatment II: wounds managed using ASR + external AMF, with daily replacement. More experimental details and methods are provided in the supporting information.

### Statistical Analysis

Each experiment was performed at least three times. All of the data were expressed as the mean ± standard deviation (SD). The results of significant differences were analyzed by GraphPad Prism, Origin, Image J, and SPSS (IBM SPSS statistical 19) software. A 95% confidence level was applied to all analyses, employing two‐tailed tests. Statistical significance was defined as follows: ^*^ *p* < 0.05, ^**^ *p* < 0.01, ^***^ *p* < 0.001.

## Conflict of Interest

The authors declare no conflict of interest.

## Author Contributions

W.Z. contributed to methodology, validation, formal analysis, investigation, data curation, and writing of the original draft. P.X. contributed to methodology and investigation. Y.G. handled writing, review, and editing. Y.H. was involved in investigation and data curation. G.Z. worked on formal analysis and data curation. Y.S. and Y.C. contributed to methodology and investigation. C.W. focused on formal analysis and investigation. W.Z., Y.L., and H.Y. contributed to conceptualization, methodology, writing, review and editing, project administration, and funding acquisition.

## Supporting information



Supporting Information

Supplemental Movie 1

Supplemental Movie 2

Supplemental Movie 3

Supplemental Movie 4

Supplemental Movie 5

Supplemental Movie 6

## Data Availability

The data that support the findings of this study are available from the corresponding author upon reasonable request.
