# Supplementary material for: Amoeba‐Inspired Soft Robot for Integrated Tumor/Infection Therapy and Painless Postoperative Drainage
Source: Adv Sci (Weinh). 2024 Nov 4;11(47):2407148. doi: 10.1002/advs.202407148 (PMC11653624; doi:10.1002/advs.202407148)
Supplement: Supplementary file 1 — Supporting Information [file ADVS-11-2407148-s006.docx]

**Supplementary Information**

**Amoeba-inspired soft robot for** **integrated** **tumor/infection therapy and** **painless postoperative drainage**

Wanyi Zhou ^1^, Peizheng Xiong ^2^, Yiman Ge ^2^, Yuhan He ^1^, Yue Sun ^3^, Gang Zhang ^4^, Yifan Chen ^1^, Chunhui Wu ^1^, Wei Zhang ^1 *^, Yiyao Liu ^1, 2, 5, 6 *^, Hong Yang ^1 *^

*^1^ Department of Orthopedics, Sichuan Provincial People's Hospital, School of Life Science and Technology, University of Electronic Science and Technology of China, Chengdu 610054, Sichuan, P.R. China*

*^2^ TCM Regulating Metabolic Diseases Key Laboratory of Sichuan Province, Hospital of Chengdu University of Traditional Chinese Medicine, No. 39 Shi-er-qiao Road, Chengdu 610072, Sichuan, P. R. China*

*^3^ School of Mechanical and Electrical Engineering, Chengdu University of Technology, Chengdu 610059, Sichuan, P. R. China*

*^4^ Department of Oncology, Chengdu Second People 's Hospital, Chengdu 610072, Sichuan, P. R. China*

*^5^ Department of Urology, Deyang People's Hospital, Deyang 618099, Sichuan, P. R. China*

*^6^ Chongqing Engineering Laboratory of Nano/Micro Biomedical Detection Technology, Chongqing University of Science and Technology, Chongqing 401331, P. R. China*

**^*^Corresponding authors**

E-mail addresses: zhangweigk@med.uestc.edu.cn (W. Zhang), [liuyiyao@uestc.edu.cn](mailto:liuyiyao@uestc.edu.cn) (Y. Liu), or [yanghongyh@uestc.edu.cn](mailto:yanghongyh@uestc.edu.cn) (H. Yang)

**1. Experimental** **Section**

***1.1 Materials***

N-hydroxysuccinimide (NHS), 1-(3-Dimethylaminopropyl)-3-ethylcarbodi-imide hydrochloride (EDC·HCl), ethylene glycol, urea, and polyvinylpyrrolidone (PVP, *M_W_* = 24000 Da) were supplied by Shanghai Macklin Biochemical Co., Ltd. (China). Methyl blue(MB), potassium permanganate (KMnO_4_), 1,1-diphenyl-2-picrylhydrazyl (DPPH), 2, 2′-azino-bis(3-ethyl-benzothiazoline-6-sulfonic acid) (ABTS), 2-Phenyl-4,4,5,5-tetrame-thylimidazoline-3-oxide-1-oxyl (PTIO), and 2,20-Azobis (2-amidinopropane) dihydro-chloride (AAPH) were purchased from Shanghai Aladdin Bio-Chem Technology Co., Ltd. (China). Hydrogen Peroxide(H_2_O_2_) Content Assay Kit was from Beijing Solarbio Science & Technology Co.,Ltd. Cell Counting Kit-8 (CCK-8), 2′,7′-dichlorofluorescin diacetate (DCFH-DA), Annexin V-FITC/PI dead cell apoptosis kit, and dihydroethidium (S0063) were supplied by Beyotime Biotech. Co., Ltd. (China). Tris (4,7-diphenyl-1,10-phenanthroline)ruthenium (II) dichloride ([Ru (dpp)_3_]Cl_2_ ) was purchased from Sigma Co., Ltd. (USA). Acridine orange- ethidium bromide (AO-EB) double fluorescence staining kit was from Shanghai Maokang Biotechnology Co., Ltd. Collagen I antibody (GB11011-3) was obtained by Servicebio Co., Ltd. Rabbit anti-TNF-α antibody (bs-2150R) and rabbit anti-TNF-α antibody (bs-10802R) were supplied by Beijing Bioss Biotechnology Co., Ltd (China).

***1.2 Synthesis of HA-PBA***

2 g of HA was dissolved in 200 mL of deionized water, according to the molar ratio of HA: PBA: EDC: NHS = 1: 2: 2.5: 2.5; PBA, EDC, and NHS were added to the HA solution. The mixture was stirred for 24 h at 4℃ and dialyzed with ultrapure water for 3 days. HA-PBA was obtained after freeze-drying.

***1.3 Synthesis of FPC Nps***

***Synthesis of Fe_3_O_4_:*** 4 mmol FeCl_3_·6H_2_O was dissolved in 40 mL of ethylene glycol, then 1.33 mmol PVP and 20 mmol urea were added into the mixture solution. The uniformly mixed solution was transferred to a high-temperature autoclave and heated to 200℃ for 12 h. The product was washed with deionized water to obtain Fe_3_O_4_ nanoparticles (NPs).

***Synthesis of FP Nps:*** Fe_3_O_4_ NPs (100 mg) and dopamine hydrochloride were added to the tris buffer (200 mL) according to the mass ratio 1:1, reacted for 6 h under rapid stirring to obtain FP NPs.

***Synthesis of FPC Nps*:** PVP (2 g) was dissolved in an aqueous solution containing CuCl_2_·2H_2_O (20 mL, 0.01 M). Then, 10 mg FP NPs, NaOH (20 mL, 0.02 M), and H_2_O_2_ (400 μL) were added to the above mixture. After stirring for 3 h, FPC NPs were collected by a magnet.

***1.4 Characterization*s**

***Structure and constitutes of components:*** The size, shape, and elemental composition of NPs and Amoeba-inspired soft robot (ASR) were characterized using a JEM-2010 transmission electron microscope (TEM) with an energy-dispersive X-ray spectrometer (EDS) (JEOL, Tokyo, Japan), scanning electron microscope (SEM; Carl Zeiss, Oberkochen, Germany), and X-Ray Photoelectron Spectroscopy (XPS, Thermo Kalpha). The magnetic properties of nanoparticles were measured using an MPMS3 superconducting quantum interference device (SQUID) magnetometer (Quantum Design, San Diego, CA, USA). The size distribution and ζ potential of NPs were measured by dynamic light scattering (DLS) using a Zetasizer (NanoZS, Malvern Panalytical Instruments, UK.) The chemical structure of HA-PBA and hydrogels were confirmed by SEM, ^1^H nuclear magnetic resonance (^1^H-NMR, Bruker 400M, Germany), and Fourier-transform infrared spectroscopy (FT-IR, Thermo Fisher Scientific, Waltham, MA, USA).

***Swelling test:*** The original weight (m_0_) of prepared hydrogel sample was measured and was immersed in PBS (pH = 7.4) at 37℃. After every 0.5 h, the hydrogel was taken out, wiped surface moisture and weighed (m_t_). The swelling ratio (SR) was calculated using the following formulas:

SR = $\frac{mt-m0}{m0}\times100\%$

***Rheological test:*** The rheological properties of ASR were determined using a rheometer (Anton Paar, MCR302). Rheological testing of hydrogels includes (1) Frequency scanning of ASR (25℃，37℃, and 45℃). The scanning frequency varied from 0.1 to 100 rad/s, and the constant strain of the frequency scanning test was 1%. (3) Strain amplitude sweep tests of ASR (37℃), the constant frequency of 60 rad/s. (4) The G′ and G′′ of ASR (37℃) from alternate-step strain sweep, the constant frequency of 10 rad/s, when the alternate step strain was switched from 1% and 100%.

***Maximum deformation size test:*** The ASRs (weight 0.5 g, diameter 1 cm) were tested on various interfaces, including plastic, paper, wet pig skin, and wet pig muscle tissue. A sintered rubidium iron boron magnet (N48), with a peak surface magnetic field strength of about 800 mT, was employed to drive ASRs to deformation and movement. The maximum deformation length of the continuum body of ASR and the deformation rates were recorded. Each group of experiments was performed in triplicate with three parallel ASRs.

The deformation rate of the soft robots is shown in the formula:

Maximum deformation rate (%) = [(L_t_/L_0_] × 100%

Where L_0_ is the diameter of the initial robots, and L_t_ is the maximum long axis dimensions of the deformed robots.

***1.5 Magnetic heating efficiency in vitro***

Exposed hydrogels to the alternating magnetic field (AMF) center with a power of 660 kHz for 10 min and recorded the temperature every 30 s with a thermal camera. Measured temperature changes of ASR at different AMF powers (150 kHz, 400 kHz, and 660 kHz). Moreover, the thermal stability of ASR was tested through four AMF (660 kHz) on/off cycles.

***1.6 Self-produced H_2_O_2_ characterization***

Hydrogels were treated with KMnO_4_ solution (50 μg/mL) in different pH (6.5, 7.4 and 8.5) for 30 min. The UV−vis spectra of KMnO_4_ solution were measured from 400 to 650 nm. Further detection of the production of H_2_O_2_ was tested by H_2_O_2_ content assay kit.

***1.7*** ***POD-like activity characterization***

The POD-like activity of hydrogels at different pH and H_2_O_2_ concentrations was detected. Hydrogels were placed in a phosphate buffer containing 10 μg/mL methylene blue (MB) to record the UV–vis absorbance of the color reaction (at the wavelength of 664 nm). Furthermore, the changes in POD-like activity over time were detected.

***1.8 O_2_ generation activity***

To test the O_2_ generation of ASR, add 0.1g/mL ASR to a phosphoric acid buffer solution of different pH and record the O_2_ concentration every 5 min with a dissolved oxygen meter.

***1.9 Free radical scavenging experiment***

Refer to previous reports, 1,1-diphenyl-2-picrylhydrazyl (DPPH), 2, 2′-azino-bis(3-ethylbenzothiazoline-6-sulfonic acid) (ABTS) and 2-Phenyl-4,4,5,5-tetramethylimidazoline-3-oxide-1-oxyl (PTIO) assays were used to determine the free radical scavenging of ASR in phosphoric acid buffers of different pHs.

(1) Added ASR to a DPPH solution at 0.1g/ml in different pHs (37℃) for 1 h, and the absorbance of the DPPH solution was measured at 517 nm. The DPPH radical scavenging activity was measured as follows:

Scavenging activity = [(A_blank_ − A_sample_)/A_blank_]×100 %.

Here, A_blank_ refers to the absorbance of the DPPH solution, and A_sample_ is the absorbance of the DPPH solution after soaking the hydrogels for 30 min. (2) Mixed a 7.4 mM ABTS solution with 2.6 mM potassium persulfate (K_2_S_2_O_8_) in the dark at 25℃ for 12 h to prepare ABTS radical cation (ABTS^+^). The absorbance of the ABTS^+^ solution was 734 nm; other experimental steps and calculations were the same as above. (3) The absorbance of PTIO at 557 nm was tested; other experimental steps and calculations are the same as above.

***1.10 Tissue adhesion properties***

Adhering two pieces of pig skin tissue with 0.02g hydrogel (adhesive area 10 mm × 10 mm) and testing the sample on a tensile testing machine (INSTRON 5565, INSTRON, Norwood, MA, USA) at a speed of 10 mm/min until the two pieces of skin tissue are separated. The bonding strength is calculated based on the maximum modulus of the bonding area.

***1.11 In vitro antibacterial experiment***

The Staphylococcus aureus (S. aureus) strains were provided by Nanjing Cobioer Biosciences Co., Ltd. Immersed hydrogels in *S. aureus* suspension (OD_600_=0.5) at a concentration of 0.1g/mL (pH=6.5) and then incubated for 24 h. The temperature of ASR was maintained at about 45-50℃ (5 min) in AMF. Survival of *S. aureus* was represented by the absorbance of bacterial suspension (at the wavelength of 600 nm). The bacterial survival rate was calculated using the following formulas:

Survival of *S. aureus* (%) = [(OD _sample_ − OD _blank_)/(OD _control_ − OD _blank_)]×100%

Diluted the co-cultured bacterial suspension 10^-6^ times and coated 10 μL diluted suspension on the corresponding solid culture medium. Observed the colony count after cultivation for 24 h.

***1.12 Transcriptome sequencing of ASR-treated S. aureus***

The antibacterial activity of ASR against *Staphylococcus aureus* (*S. aureus*, 26003-5a28-1) was evaluated *in vitro*. The *S. aureus* and 0.1 g/mL ASR was co-incubated with LB medium in pH=6.5 for 8 h, then was used for transcriptome sequencing. *S. aureus* treated with only LB medium in pH=6.5 was used as a control. The library preparation and sequencing were completed by Shanghai Bioprofile Technology Co., Ltd (China). Differentially expressed genes were analyzed by the Kyoto Encyclopedia of Genes and Genomes (KEGG) enrichment and Gene Ontology (GO) enrichment.

***1.13 Biocompatibility and magnetothermal therapy in vitro***

All experimental cell lines, including mouse fibroblasts (L929 cells) and mouse cutaneous melanoma cells (B16F10 cells), were purchased from Procell Life Science&Technology Co., Ltd. To evaluate the biocompatibility of hydrogels, 0.1 g/mL hydrogels were incubated with L929 cells and B16F10 cells in the RPMI-1640 (pH=7.4) for 24 h, respectively. CCK-8 kit was used to analyze cell viability. To detect the magnetocaloric effect of ASR, 0.1g/mL hydrogels were incubated with B16F10 cells in RPMI-1640 (pH=6.5) for 12h. Cells covered with hydrogels were exposed to AMF (660 kHz) for 10 min and continued incubation for 12h. Cells were analyzed by CCK-8 kit, acridine orange-ethidium bromide (AO-EB) staining kits, and FACS canto II flow cytometry (BD Biosciences, USA).

***1.14 Detection of apoptosis by flow cytometry***

For groups with magnetothermal treatment, B16F10 cells and 0.1g/mL hydrogels were treated with alternating magnetic field (AMF, 660 HZ) for 10 min followed by co-culture for 24 h in RPMI-1640 medium (pH=6.5). At the end of 24 h, flow cytometry was performed on 15,000 cells in each group by the Annexin V-FITC Apoptosis Detection Kit (annexin V–FITC/PI). For groups without magnetotherapy, the above experimental steps are also followed, but these cells are not exposed to AMF.

***1.15 The ROS production/scavenging of ASR in vitro***

To test the ROS production, B16F10 cells and 0.1g/mL hydrogels co-cultured in RPMI-1640 medium (pH=6.5) for 12 h; to estimate ROS scavenging, L929 cells were treated with 100 μM AAPH in RPMI-1640 medium (pH=7.4) for 30 min to structure intracellular ROS elevation model, then, L929 cells and 0.1g/mL hydrogels co-cultured in pH=7.4 for 12 h. Finally, B16F10 and L929 cells were stained by the fluorescent DCFH-DA probe of ROS assay kit, respectively.

***1.16 Intracellular O_2_ measurement***

The L929 cells were cultured with RPMI-1640 medium (pH=7.4) in an Anaeropack anaerobic system previously for 24 h to establish a cellular hypoxia model. Then, L929 cells and hydrogels were incubated in the anoxic environment for 12 h, and oxygen generation was detected by [Ru (dpp)_3_]Cl_2_ (luminescent oxygen sensor).

***1.17 Animal wound healing test***

All experimental animals were obtained from GemPharmatech Co., Ltd. The animal experiments of this study followed the guidelines set of experimental animal management and welfare ethics of University of Electronic Science and Technology of China (accreditation number: 1061423022725123).

***Full-thickness excision infected wounds:*** A wound of d = 8 mm was created in the dorsal area of mice. Inoculated *S. aureus* suspension (OD_600_=0.5, 100 μL) at the damage, then covered the wound with different dressings. A portable pH acidity meter glass plane was used to click to detect the pH of wounds. The calculation of wound closure is shown in the formula:

Wound heating (%) = [(A_0_ − A_t_)/A_0_] × 100%

Where A_0_ is the initial area, and A_t_ is the wound area on day t.

***Full-thickness excision wounds with incompletely resected tumors:*** 100 μL B16F10 suspension (2×10^6^ cells/mL) was injected into the back of the mice to establish the subcutaneous tumor-bearing models. After the tumor volume reached ~100 mm^3^, an 8 mm diameter full-thickness excision was performed at the tumor site, and part of the tumor tissue was excised. Tumor tissue with a volume of 40 mm^3^ was intentionally left at the wound site to ensure that the tumor in the control group recurred. Mice were randomly divided into 4 groups (n=6 per group). The control group dressed the wounds with gauze; the treatment Ⅰ group dressed the wounds with 0.5 g ASR; the treatment Ⅱ group dressed the wounds with 0.5 g ASR; treated with AMF to maintain the wound temperature 45-50℃ 5 min every 48 h; the treatment Ⅲ group: same treatment as the treatment Ⅱ group, and change dressings every 48 h. A portable pH acidity meter glass plane was used to detect the pH of wounds and tumors respectively. The calculation of wound closure is the same as above, and the calculation of tumor recurrence is shown in the formula:

Tumor recurrence (%) = [(A_t_ – A_0_)/A_0_] × 100%

Where A_0_ is the initial tumor area, and A_t_ is the tumor area on day t.

***Infected sinus tract wounds:*** Created a tunneling wound with a diameter of 8 mm and a depth of 20 mm on the rabbit thigh and inoculated *S. aureus* suspension (OD_600_=0.5, 100 μL) at the damage. After 24 h, When yellow-white purulent secretions and other signs of infection appear in the tunneling wound, it is considered a successful establishment of an infection tunneling wound model. Rabbits weighing 4-5 kg were divided into 2 groups (n=4 per group): the treatment Ⅰ group: care wounds by clinical procedures. (1) clean the wound with iodophor; (2) fill the wound with iodine gauze to remove pus; (3) change dressing daily. The treatment Ⅱ group: care wounds by 1 g ASR. Specifically, fill the wound with ASR to absorb pus and change a new ASR after daily magnetothermal treatment (maintain the wound temperature 45-50℃, 5 min). On day 5, compared with the untreated group, purulent secretions in wounds of the treatment I and II groups were significantly reduced, meeting clinical suture standards, so sutures were performed. The suture surgery follows clinical operating guidelines and adopts a layer-by-layer suture method. The residual ASR at the wound in the treatment II group was also sewn into the deep wound. the calculation of the weight of ASR is shown in the formula:

Weight of ASR (%) = (A_t_/A_0_) × 100%

Where A_0_ is the initial weight of ASR, and A_t_ is the weight of ASR that crawled out of the wound on day t.

***1.18 Histological and pre-healing factors analysis***

Wound area tissues of each group were acquired during the healing process and analyzed by H&E staining and immunofluorescence staining to clarify the wound healing mechanism. Studied pre-healing factors, including tumor necrosis factor (TNF-α) and collagen I (Col-Ⅰ).

**2. Supplementary Figures**

***2.1*** ***Synthesis and characterization of amoeba-inspired soft robot (ASR)***


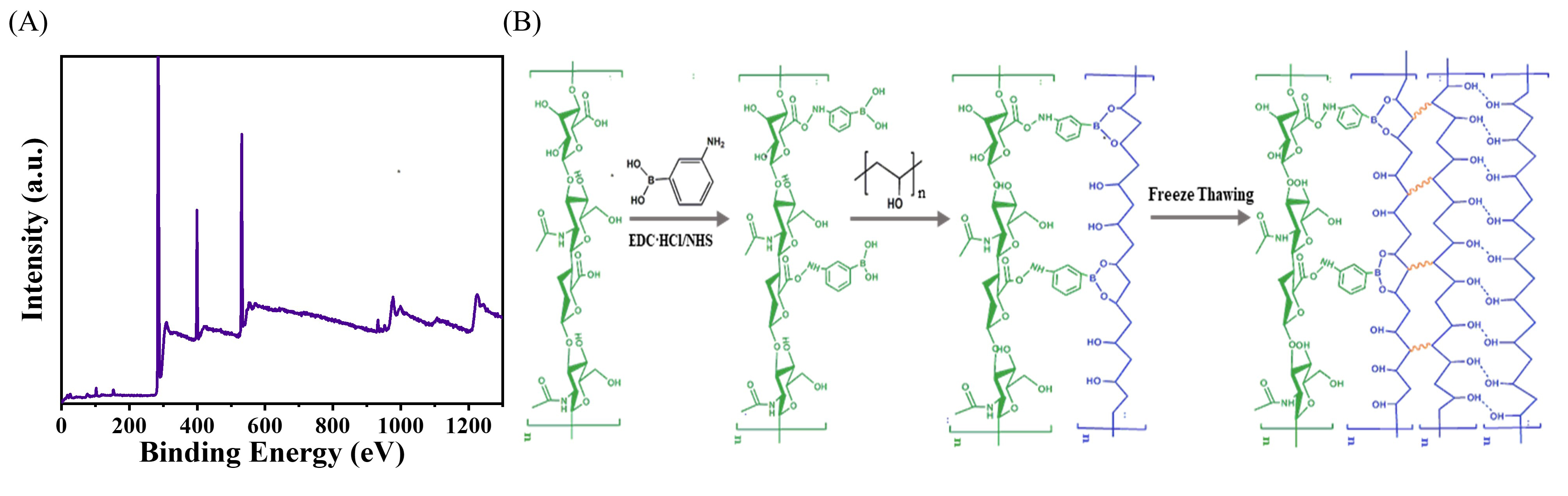


**Figure S1.** (**A**) High-resolution XPS spectra of FPC Nps. (**B**) Synthetic route of the hydrogel matrix of ASR.

***2.2 Deformation and controlled manipulation of ASR***


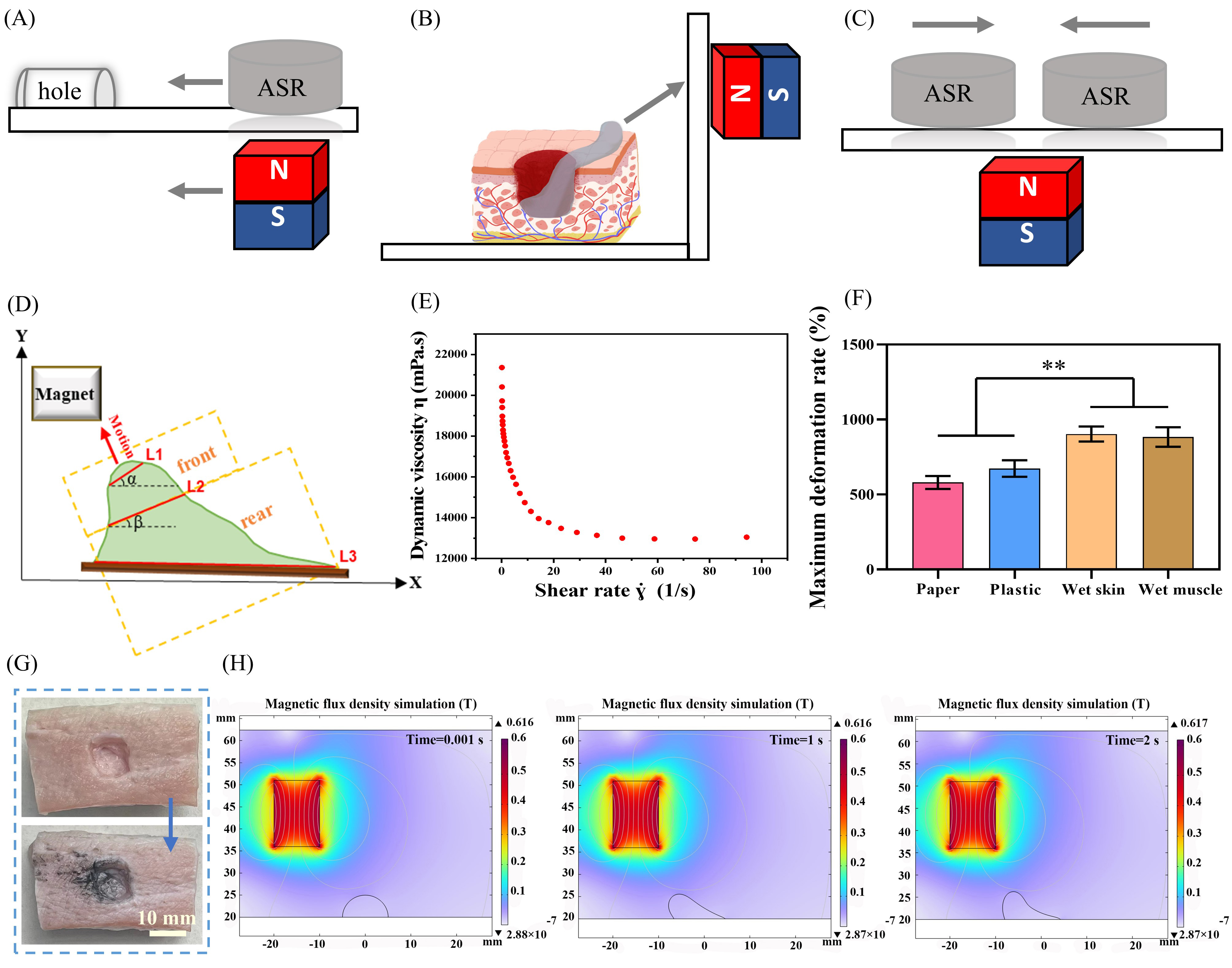


**Figure S2.** (**A**) Schematic diagram of magnet guiding ASR into the hole. (**B**) Schematic diagram of magnet-guided ASR crawling out of the wound. (**C**) Schematic diagram of magnet-guided ASR self-healing. (**D**) Mechanical analysis of formation in ASR. (**E**) Dynamic viscosity test (rotation mode). (**F**) The maximum deformation rate of the ASR at different interfaces (n = 3). (**G**) A small part of the robot remained in place during crawling.(**H**) The effect of magnet position and magnetic flux density on ASR deformation and displacement.

Figure S2D illustrates the mechanical analysis of the formation in ASR. The front and rear of ASR are distinguished based on whether it is exposed to magnetic forces. At the front of ASR, the driving force is the directional magnetic force $F_{fx}$ on the x-axis. The resistance consists of $F_{e}$, the elastic force due to ASR deformation (own cohesion force), and $f_{front}$, the adhesion force between ASR and the horizontal substrate. The net force at the front of ASR is given by:

$F_{front}=F_{fx}-F_{e}-f_{front}=\int\nabla\left( H\cdot B_{x} \right)dV-E\varepsilon A-\gamma cos\theta_{adv}\cdot L_{1}$ (1)

Where $E$ is the elastic modulus of ASR, $\varepsilon$ is the tension rate of ASR, $A$ is the interface (cross-sectional area) between the front and rear part of the robot, $\theta_{adv}$ is the advancing contact angle of the droplet on the substrate, $L_{1}$ is the length of forward moving contact line in the front of ASR, and $\alpha$ represents the angle between $L_{1}$ and the substrate. In the presence of a magnetic field, ASR can deform and form a protrusion at its leading edge if the magnetic force is strong enough to overcome the ASR’s resistance ($F_{front}>0)$.

The forward and backward motion of the rear of ASR are determined by the equilibrium between two distinct forces: the elastic force caused by ASR deformation ($F_{e}$) and the adhesion force between ASR and the substrate ($f_{rear}$). So, the net force at the rear of the robot can be derived as:

$F_{rear}=F_{e}-f_{rear}=E\varepsilon A+\gamma\left( cos\theta_{rec}-cos\theta_{adv} \right)\cdot L_{3}-cos\theta_{adv}\cdot L_{2}$ (2)

where $\theta_{rec}$ is the receding contact angle of ASR on the substrate, $L_{2}$ is the width of the interface (cross-sectional section) between the front and back part of the robot, $L_{3}$ is the contact line between the robot and the substrate in the rear of ASR, and $\beta$ is the angle between $L_{2}$ and the substrate. If $F_{rear} > 0$, the robot will move while deformation at the front.

The simulations of ASR in this work were carried out using the COMSOL Multiphysics software (Version 6.0, COMSOL Inc). The method simulates a uniform magnetic field produced by a magnet cylinder. The ASR was assumed incompressible and modeled as a magnetic Newton fluid. The laminar blood flow in a single-phase fluid (SPF) model is considered the surrounding environment. Furthermore, the Smooth Particle Hydrodynamics method models ASR to study and guide the magnetic control of their deformation and motion. The gravity was considered during the simulation, and all walls were defined as no-slip boundaries. No Currents Interface method simulates the uniformed magnetic field created by the cylinder magnet (size of 10×15mm^3^).

Since the magnetic fluid is incompressible, so the Navier-Stocks Equation for ASR can be expressed as:

$\rho\frac{\partial u}{\partial t}=-\nabla p_{m}+v\left( B, \gamma\right)\nabla\cdot\left( \nabla u+\left( \nabla u \right)^{T} \right)+\rho g$ (3)

Where $\rho$ is the magnetic fluid density, $u$ is the velocity of ASR, $v$ is the viscosity depending on the magnetic induction intensity $B$ and shear rate $\gamma$, and $g$ is the gravity.

The pm pressure is mainly produced by magnetism, which can be expressed as:

$p_{m}=\frac{\partial}{\partial V}E_{m}$ (4)

Where $V$ is the volume of ASR, $E_{m}$ is the magnetic energy, which can be expressed as:

$E_{m}=\int\frac{B\cdot H}{2}\partial V$ (5)

The magnetic induction intensity $B$ can be expressed using a constant value with the magnitude of the external magnetic field $H$.

***2.3 Whole transcriptome RNA sequencing of ASR***


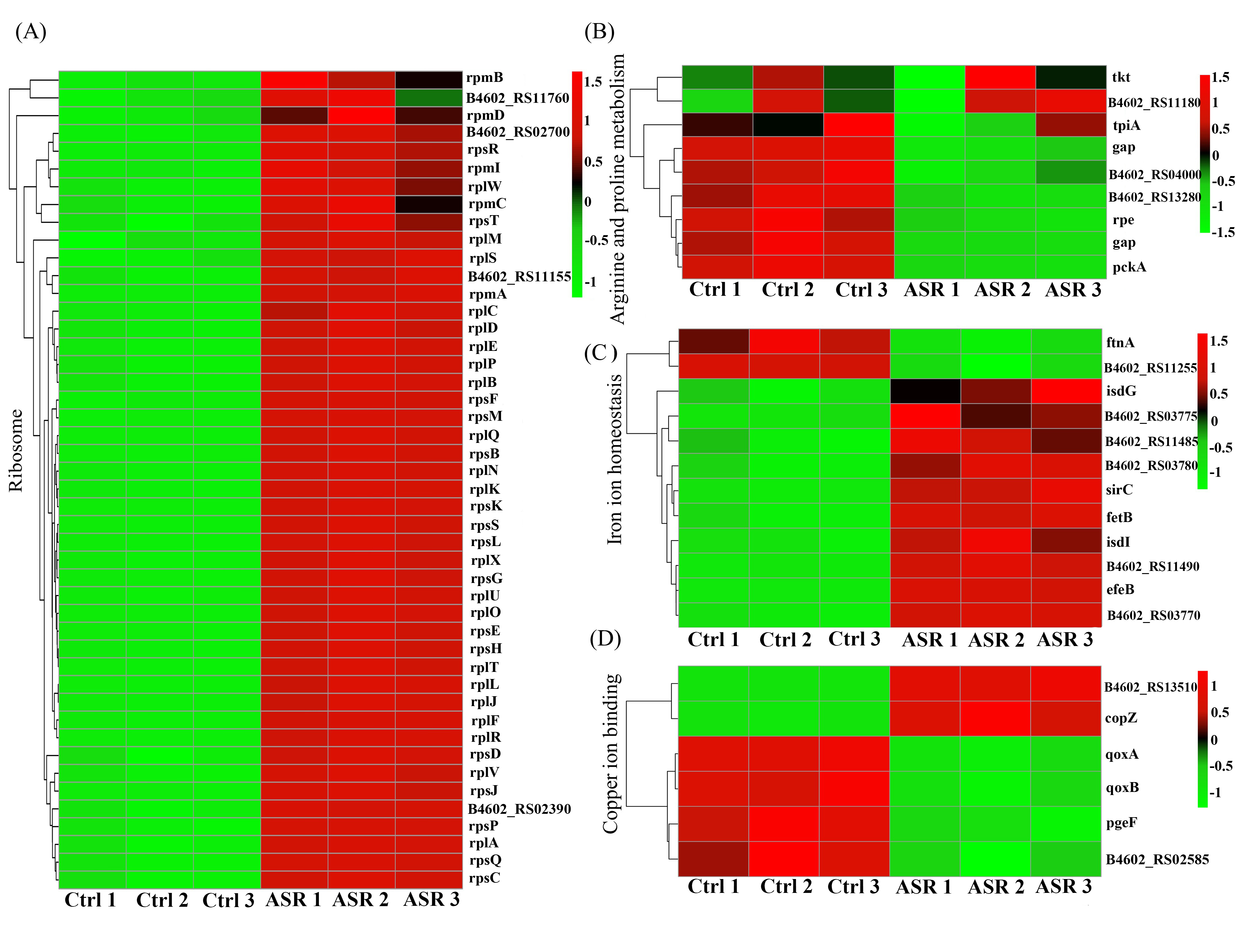


**Figure S3.** (**A**) Heatmap of genes associated with ribosome (n = 5). (**B**) Heatmap of the genes related to arginine and proline metabolism (n = 5). (**C**) Heatmap of genes related to iron ion homeostasis (n = 5). (**D**) Heatmap of the genes related to copper ion binding (n = 5).

***2.4 Cytotoxicity of ASR***


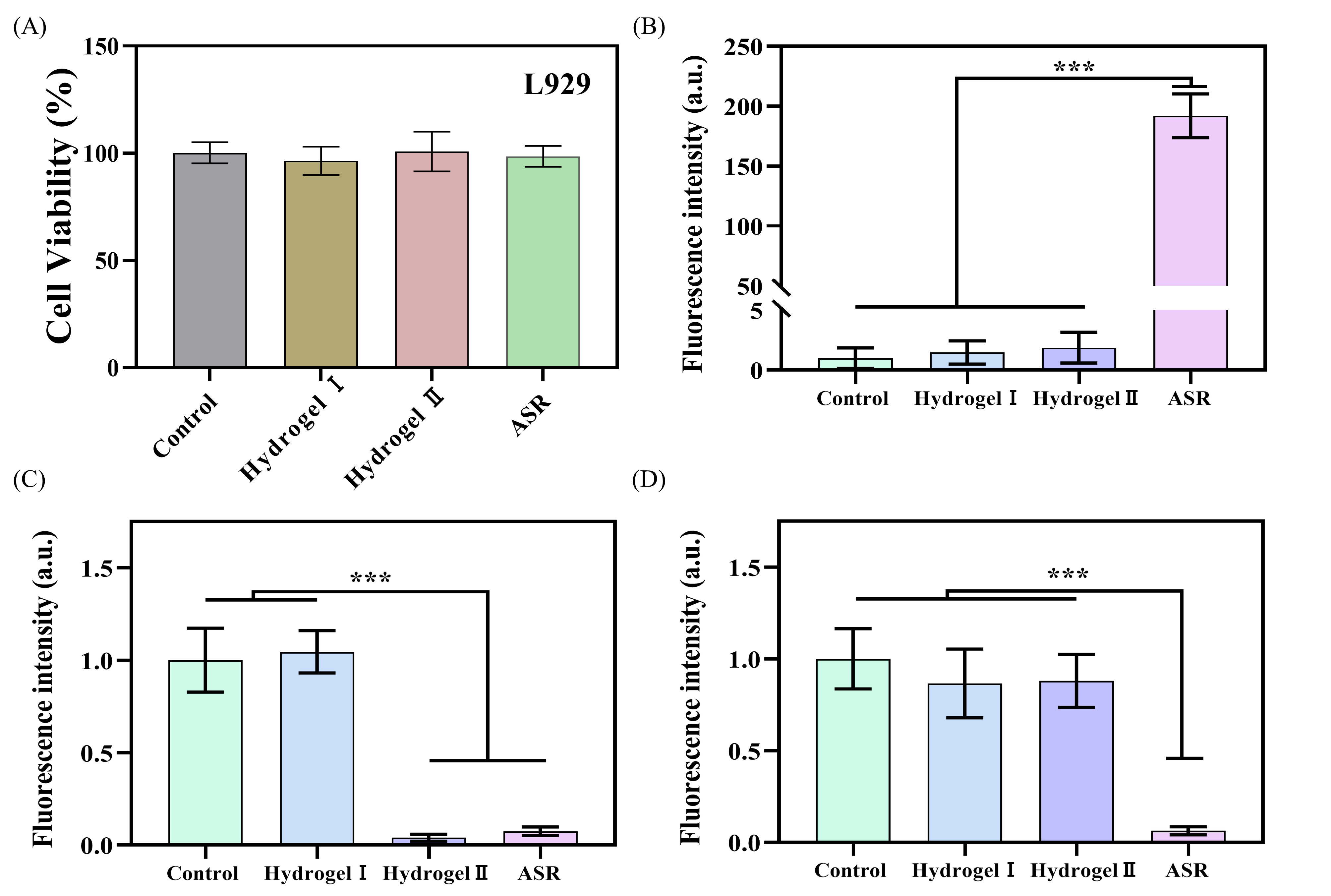


**Figure S4.** (**A**) Effects of different hydrogels on cell viability of L929 cells at neutral pH (n = 3). (**B-D**) Fluorescence intensity statistical results of the ROS in B16F10 cells, the ROS in L929 cells, and the O_2_ production in anoxic L929 cells (n = 3). Note: Scale bar: 100 μm. Note: Hydrogel I: HBP hydrogel (hydrogel matrix of ASR); Hydrogel II: HBP hydrogel containing FP Nps.

***2.5 Healing of infected skin defect wounds in vivo***


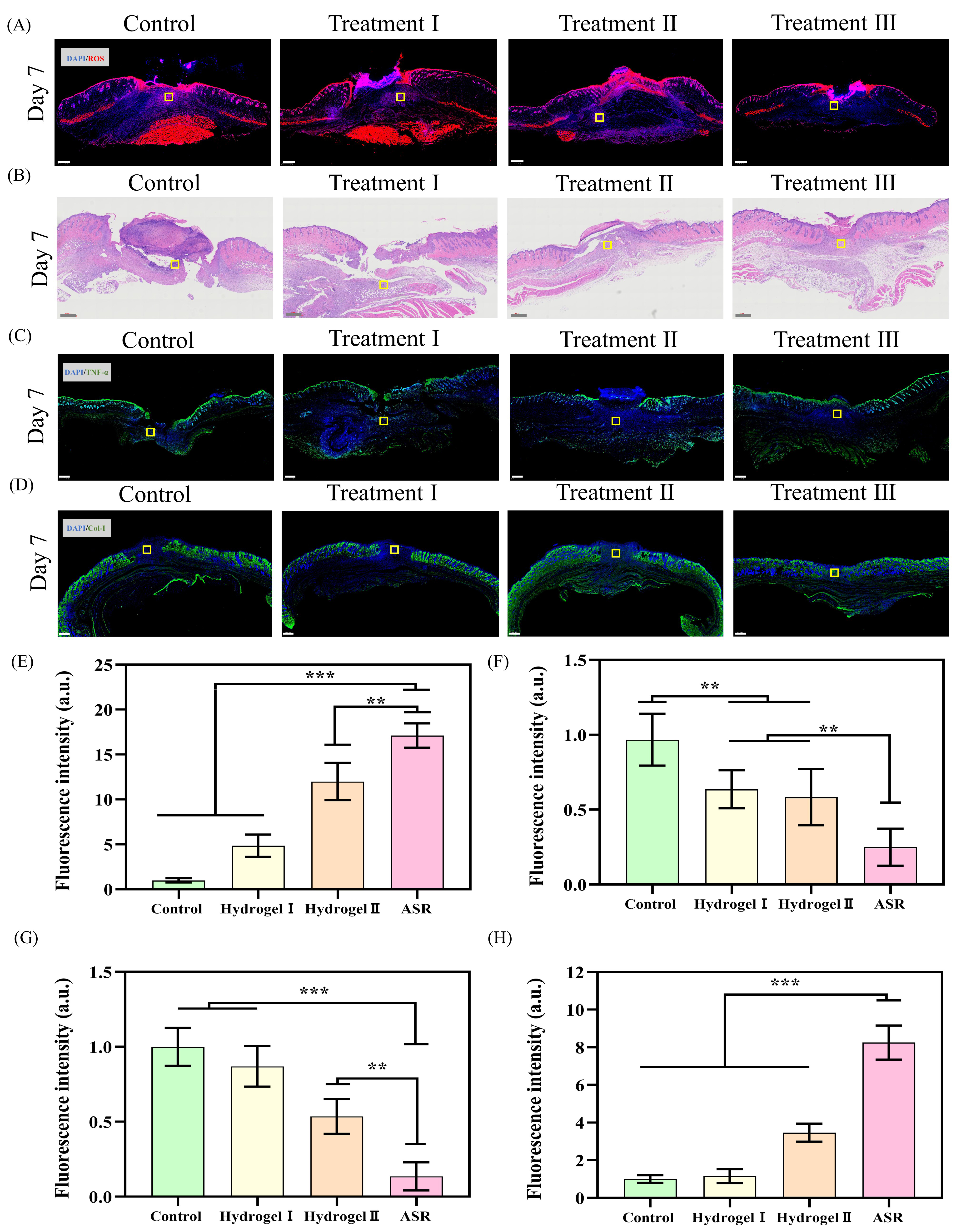


**Figure S5.** (**A**) Images of ROS content of wounds on day 7. (**B**) Images of H&E staining of wounds on day 7. (**C**) Images of immunofluorescence staining of TNF-α of wounds on day 7. (**D**) Images of immunofluorescence staining of Col-Ⅰ of wounds on day 12. Note: scale bars: 500 µm. H&E stain and immunofluorescence images of wounds in Figure 7 are the local magnification of tissues in the yellow box in Figure S5. The grouping scheme was provided in the materials and methods. (**E**) Statistics of ROS levels in bacteria collected from the wound site on day 5 (n = 3); scale bar: 100 μm. (**F-H**) Immunofluorescence intensity statistical results of ROS, TNF-α, and Col-Ⅰ at the wound site (n = 3); scale bar: 40 μm.

***2.6 Healing of wounds after tumor resection in vivo***


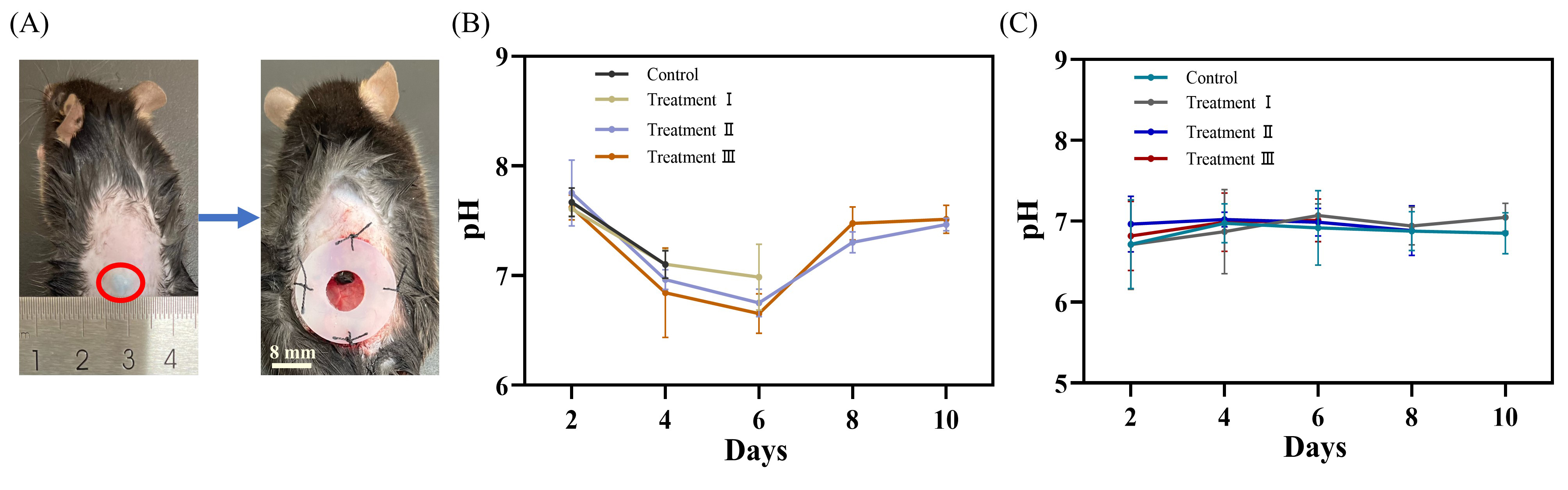


**Figure S6.** (**A**) Establishing the wound model: removed 90% of melanoma and formed skin defect wounds. (**B**) The pH of the skin defect wounds (pH of wounds was no longer measured when the wound size was too small) (n = 3). (**C**) The pH of melanoma (pH of tumors was no longer measured when the tumor size was too small) (n = 3).

***2.7 Healing of*** ***infected tunneling wounds in vivo***


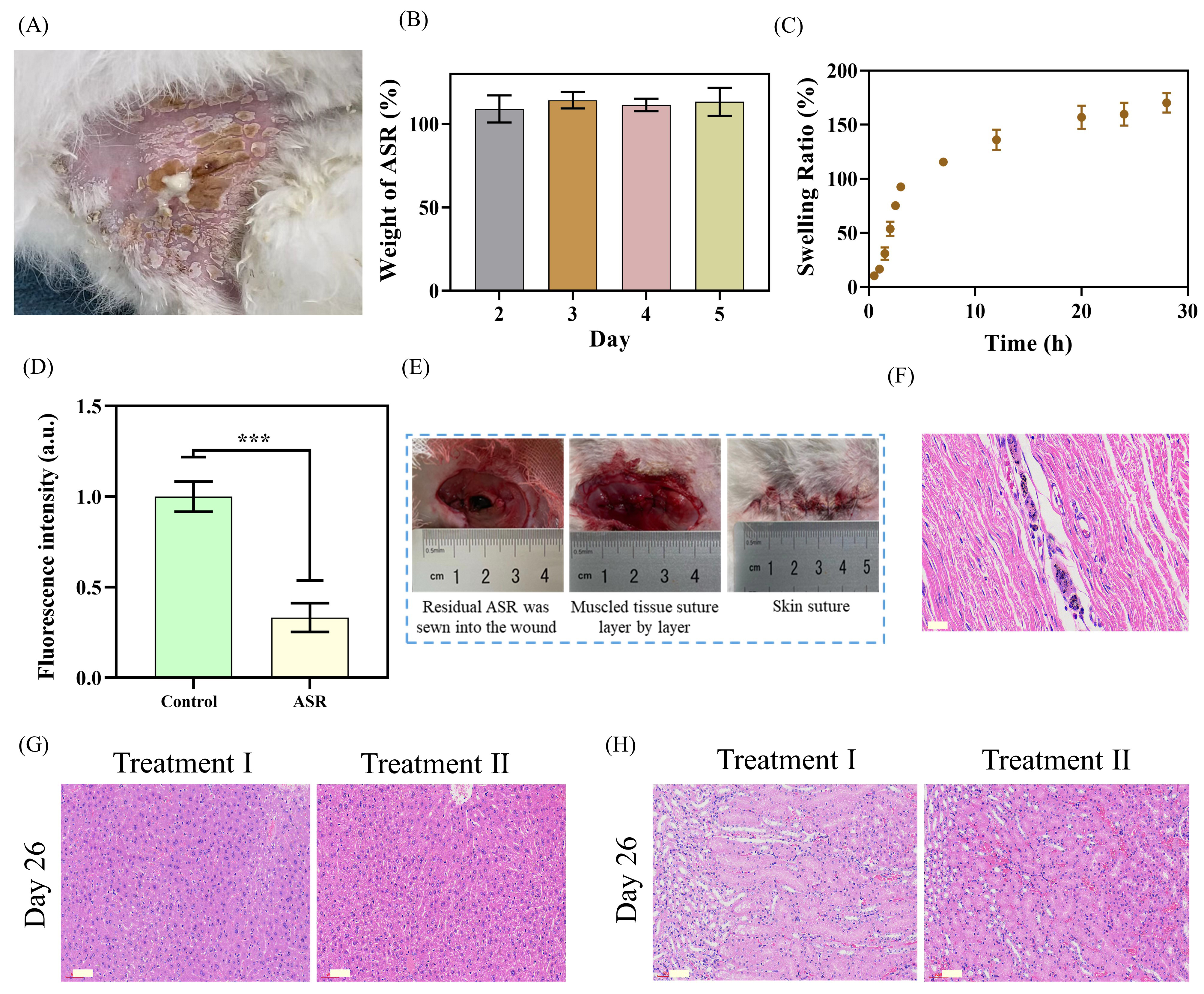


**Figure S7.** (**A**) Wound condition of rabbits in the untreated group on day 5 (scale bars: 8 mm). (**B**) The weight of ASR after crawling out of the wound (n = 3). (**C**) Swelling ratio of ASR at 37℃ (n = 3). (**D**) Immunofluorescence intensity statistics of TNF-α at the wound site on day 26 (n = 3); scale bar: 100 μm. (**E**) The suture process of infected tunneling wounds. (**F**) Residual ASR was engulfed by multinucleated cells (scale bars: 30 µm). (**G**) Images of H&E staining of livers on day 26 (scale bars: 60 µm). (**H**) Images of H&E staining of kidneys on day 26 (scale bars: 60 µm).
